# Capstone Simulation: A Multipatient Simulation for Senior Emergency Medicine Residents

**DOI:** 10.15766/mep_2374-8265.11361

**Published:** 2023-11-09

**Authors:** Caitlin Schrepel, Anne K. Chipman, Ross Kessler, Crystal Phares, Elizabeth Rosenman

**Affiliations:** 1 Assistant Professor and Assistant Program Director, Department of Emergency Medicine, University of Washington School of Medicine; 2 Assistant Professor and Assistant Director of Quality Improvement, Department of Emergency Medicine, University of Washington School of Medicine; 3 Assistant Professor and Ultrasound Fellowship Program Director, Department of Emergency Medicine, University of Washington School of Medicine; 4 Chief Resident, Department of Emergency Medicine, University of Washington School of Medicine; 5 Associate Professor and Director of Simulation, Department of Emergency Medicine, University of Washington School of Medicine

**Keywords:** Advanced Cardiac Life Support, Advanced Trauma Life Support, Delivering Bad News, Task-Switching, Emergency Medicine, Simulation

## Abstract

**Introduction:**

Emergency medicine (EM) trainees must learn to manage multiple patients simultaneously using task-switching. While prior work has demonstrated that multipatient scenarios can be an effective teaching tool for task-switching, few studies have shown how simulation can be used to assess residents' ability to manage multiple patients effectively. The goal of this curriculum was to provide a formative assessment of core EM skills by employing a series of simulations designed to require frequent task-switching.

**Methods:**

This exercise consisted of three simulation scenarios running in sequence. The first scenario involved medical resuscitation and advanced cardiac life support, the second required learners to manage two patients involved in a trauma using advanced trauma life support, and the final scenario tested learners' ability to communicate bad news. Faculty observers used scenario-specific checklists to identify gaps in content knowledge, communication skills, and task-switching abilities during reflective debriefs. These checklists were analyzed to identify trends. All participants were sent a postsession evaluation. Items omitted by >50% of participants were flagged for review.

**Results:**

Flagged items included asking for finger-stick glucose, verbalizing a backup intubation plan, specifying type of blood products, and asking for team input. Nine of 12 participants completed the postsession evaluation, noting that they agreed or strongly agreed the simulation was relevant and promoted reflection on task-switching skills.

**Discussion:**

This simulation provides educators with a tool to facilitate reflective feedback with senior EM learners regarding their core resuscitation, leadership, and task-switching skills and could be further adapted to promote deliberate practice.

## Educational Objectives

By the end of this activity, learners will be able to:

1. Identify their strengths and deficiencies in advanced cardiac life support and advanced trauma life support management.
2. Recognize strengths and areas for improvement in airway management skills.
3. Identify strengths and areas for improvement in skills of breaking bad news.
4. Describe strengths and areas for improvement in their leadership and communication skills.
5. Identify strengths and weaknesses in their ability to task-switch between scenarios.

## Introduction

Managing multiple patients simultaneously is a critical skill for emergency medicine (EM) trainees to master prior to graduation from residency.^1^ EM ACGME milestones state that residents approaching graduation should be able to “employ task-switching in an efficient manner to manage the emergency department [ED]”^2^ This skill is crucial given the need to manage a large number of patients simultaneously with various levels of acuity while addressing interruptions.^3^ Although clinical education is a key to mastering task-switching, simulation can also be used to effectively teach and assess the skills needed for managing multiple patients.^4–7^ Previously, simulations have been created to mimic the task-switching skills needed for clinical scenarios such as rapid ECG interpretation^8,9^ and the evaluation of shock states.^10^ While these curricula focus on specific clinical scenarios, simulation has also been used to recreate the clinical diversity an EM physician might experience on shift.^6,7^ For example, a curriculum in *MedEdPORTAL* presents a simulation-based session that requires PGY 2 residents to task-switch between diverse patient scenarios such as bronchiolitis and Ebola exposure.^7^

Although this prior work has demonstrated that multipatient scenarios are well liked by learners^6,7^ and can be used as a tool in teaching task-switching,^4^ there is more to learn about how simulation can be used to assess residents' ability to task-switch effectively. Task-switching skills are generally assessed through direct observation or through the use of tools such as the mini-CEX.^1,11,12^ However, a recent study demonstrated that simulation can play a role in assessing task-switching skills,^5^ suggesting educators should continue to learn more about the utility of simulation for this purpose. Therefore, the goal of this curriculum was to provide a formative assessment of core EM skills through a series of simulations specifically designed to require frequent task-switching. Debriefing sessions with faculty observers were used to promote self-reflection and to offer guidance on further skill development prior to graduation.

## Methods

### Development

This simulation was completed as a required exercise by PGY 3 residents at our 4-year EM program. It consisted of three sequential scenarios. Scenario 1 (Appendices A–D) included an adult patient with cardiac arrest, requiring advanced cardiac life support (ACLS), airway, and ST segment elevation myocardial infarction (STEMI) management (Appendix A). Scenario 2 (Appendices E–I) involved one adult and one pediatric patient from a motor vehicle collision, requiring advanced trauma life support (ATLS) management (Appendix E). Finally, scenario 3 (Appendices J–L) required the trainee to deliver bad news to a family member of the patient from scenario 1 who had subsequently died (Appendix J). We selected this content to assess core EM competencies that are generally high acuity and high stress. The assessment checklists for each scenario (Appendices D, I, and L) were developed to highlight any gaps in participant skills and knowledge in order to facilitate formative feedback and discussion during the debrief. These checklists were developed by our team with expertise in EM and simulation and included relevant guidelines pertaining to ACLS, ATLS, airway management, and delivering bad news.^13–15^ We chose to include many detailed aspects of patient care in these checklists with the goal of providing participants the opportunity to reflect on aspects of care they might often overlook in preparing for independent practice. The checklists underwent an iterative design process, with revision guided by faculty review and previous course implementation over several years prior to the versions presented here. This project was deemed exempt by the University of Washington Institutional Review Board (STUDY00015294).

### Equipment/Environment

The capstone exercise was held in the WWAMI Institute for Simulation in Healthcare. Scenario 1 was set in a simulated ED resuscitation bay. We used a high-technology patient simulator, and vital signs were displayed on a monitor. The full setup and available equipment are described in Appendix B. Learners could request diagnostic images including electrocardiograms, chest radiographs, and laboratory results during the simulation (Appendix C). Scenario 2 was also set in a simulated ED with a partition to represent two ED exam rooms. For the adult patient, we used a high-technology patient simulator. The manikin was clothed with garments precut on the sides for easy removal. For the pediatric patient, we used a similar high-technology simulator with the voice provided by the overhead microphone in the room. The setup details and available equipment are described in Appendix F. Laboratory results, chest radiographs, and ultrasounds were available for learners to request during the scenario (Appendices G and H). Scenario 3 was held in an office with two chairs and a standardized participant (SP) following scenario prompts (Appendix K).

### Personnel

Personnel for scenario 1 included a simulation technician, an EM faculty instructor, and four embedded SPs to play the roles of nurse, paramedic, medical assistant, and respiratory therapist (Appendix B). Scenario 2 personnel included another simulation technician, EM faculty instructor, and three embedded SPs to play the role of two nurses and one medical assistant (Appendix F). The faculty instructor played the voice of the pediatric patient. Scenario 3 involved a paid SP acting as the patient's father. This SP was recruited through our medical school SP program and had extensive prior training in similar scenarios. He was prepared for this session through one-on-one instruction with EM faculty. All other embedded SPs helped on a volunteer basis. There was no overlap in staffing the scenarios in order to allow them to run simultaneously using a staggered start. Additionally, three different EM physicians functioned as dedicated observers, following individual residents through all three scenarios, completing the assessment checklists, and facilitating the individual debriefs with each resident. Prior to the session, this team prepared by reviewing all materials for a scenario. Faculty observers reviewed the assessment checklists in advance, but there was no specific rater-training.

### Implementation

We implemented the simulation during a single 8-hour day. Resident start times were staggered throughout, with each resident progressing sequentially through scenarios 1, 2, and 3. We used a schedule to maintain organization (Appendix M). The faculty observer watched from a nearby control room using live streaming and entered towards the end of each scenario, playing the role of the charge nurse with specific prompts to help the participant transition to the next scenario.

Prior to scenario 1, participants received a briefing, including written and verbal reminders of the capabilities and expectations of the simulation environment. Scenario 1 (Appendix A) began with participants receiving a history from a paramedic on an adult patient who had bystander CPR after collapsing. During the scenario, the patient became hypoxemic, necessitating intubation. Following intubation, the patient lost pulses, requiring ACLS management for a ventricular fibrillation arrest. The patient regained pulses with defibrillation after two to three cycles of CPR. An electrocardiogram demonstrated an STEMI, necessitating activation of the cardiac catheterization lab.

As this patient was getting prepared for transfer to the cath lab, a faculty observer entered the room to inform the resident that they had another patient. They then arrived at scenario 2 (Appendix E) where the SP nurse informed them that emergency medical services had brought an adult male who had been involved in a highway-speed motor vehicle collision and been intubated prior to arrival. As the resident proceeded with ATLS evaluation, the primary survey revealed diminished breath sounds and chest wall crepitus on the right, and the chest radiograph demonstrated a deep sulcus sign and subcutaneous air, concerning for a pneumothorax. Approximately 3 minutes after arrival, the patient became increasingly tachycardic, hypoxemic, and hypotensive. At this same point, the second SP nurse reported the arrival of a second patient, a 6-year-old pediatric patient with ankle pain and a headache. After initial evaluation of the pediatric patient (who was stable), the first SP nurse informed the resident that the adult patient was hypotensive. At this point, the resident had to initiate blood transfusion, call general surgery, and ask for a FAST (focused assessment with sonography in trauma) exam. The FAST was positive for fluid in the right upper quadrant and lack of lung sliding on the right. The resident needed to indicate that a chest tube or needle decompression was needed and verbally walk through the procedure. During this part of the simulation, the pediatric patient continued to complain of ankle pain until pain medications were provided. Finally, after the adult patient was stabilized, the resident had to complete the evaluation of the pediatric patient and obtain an ankle radiograph.

As scenario 2 ended, the faculty observer entered as the charge nurse to tell the resident that the first patient they had seen (in scenario 1) had died in the cath lab and that a family member had arrived and was waiting for an update. The resident then entered a small room for scenario 3 (Appendix J) where an SP played the father of the patient in scenario 1.

### Debriefing

Following the three consecutive scenarios, the dedicated faculty observer debriefed the resident with the goal of getting them to reflect on EM core concepts as well as task-switching between scenarios (Appendix N). The faculty observer first asked the participant to reflect on their performance overall, followed by a more nuanced discussion of any missed checklist items. Finally, the debrief ended with a discussion on how to integrate what the participant had learned from this exercise into their clinical practice, focusing on task-switching skills. Residents also had the opportunity to participate in a delayed debrief with one of the course faculty members. This included watching audiovisual recordings of their own scenarios, reviewing the completed checklists, and discussing their performance. Video review was an optional part of the course.

### Assessment

Two assessment tools were used during this course. First, we used the critical action checklist for each scenario to facilitate the feedback process. These checklists also helped to identify any patterns among the resident group that might inform modifications to the resident curriculum. Data were collected on paper checklists as this was most convenient for the faculty observers. Each item was then entered as done or not done in an Excel spreadsheet. Faculty observers were instructed not to give credit for items if prompted. They were encouraged to take note of prompted responses to help with debriefs. Composite scores were calculated by summing the number of completed items, with maximum possible scores of 63 for scenario 1, 46 for scenario 2, and 12 for scenario 3. Descriptive statistics were used to determine the mean and standard deviation of composite scores for each scenario. We also calculated the percentage of participants who completed each checklist item. Items completed by less than 50% of participants were flagged for further review.

Second, we gathered participant feedback about the simulation using a postcourse survey (Appendix O). All questions were scored on a 5-point Likert scale (1 = *strongly disagree*, 5 = *strongly agree*). The survey was piloted with senior residents who had already completed the simulation. Data were collected and analyzed in Excel.

## Results

We implemented this simulation in September 2022 with 11 PGY 3 EM residents and one PGY 4 EM resident (who had missed the simulation the prior year) at our 4-year EM residency program. Faculty observers completed checklists for all 12 participants. Only one participant took advantage of the delayed video review. Composite scores for each scenario are presented in Table 1. Items with less than a 50% completion rate are presented in Table 2. No items in scenario 3 were missed by >50% of participants, and residents performed well on items that directly asked about task-switching.

**Table 1.**
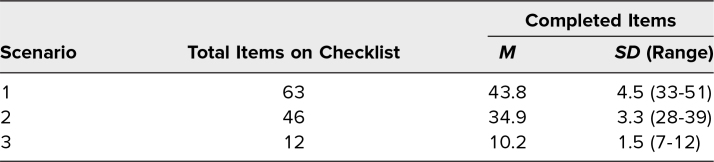
Composite Checklist Data by Scenario

**Table 2.**
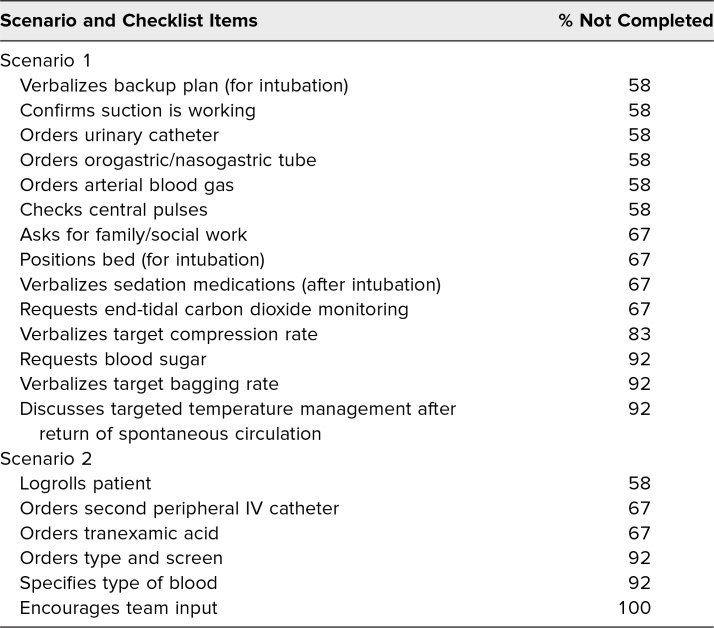
Items With >50% Rate of Noncompletion by Scenario

Nine participants (75%) completed the postsession survey (Appendix N). All respondents reported that they either agreed or strongly agreed that the simulation was relevant to their practice, was realistic, was conducted in a psychologically safe way, and promoted reflection on opportunities for improvement. Following the simulation, all respondents reported feeling confident or very confident in their ability to manage trauma patients following ATLS, task-switch between patients of different acuity levels, and self-reflect on their practice to identify gaps. Most (eight of nine) respondents reported that they felt confident or very confident in managing a patient in cardiac arrest, managing patients simultaneously, and delivering bad news, with one respondent giving a neutral response to each of these questions. In free-text responses, respondents also reported that they had learned lessons in delegating to team members, gained insight into how to switch between patients, and received tips for delivering bad news, as well as reminders about critical resuscitation skills.

## Discussion

Our curriculum demonstrates that simulation can be used as a tool for facilitating formative assessment of a senior EM resident's core knowledge and ability to manage multiple patients simultaneously while providing them with the opportunity to reflect on their task-switching skills. This builds on prior work demonstrating the utility of simulation in assessment of other competencies within medicine.^16–20^ While previous work has illustrated how simulation can be used as a task-switching assessment,^5^ our resource does so in a way that integrates skills specific to EM senior residents. This is accomplished by integrating core EM concepts including medical knowledge, patient care, communication, and team leadership within the assessment.^2^

The structured scenarios and checklists allowed both participants and faculty observers to identify specific skill and knowledge gaps, providing learners an opportunity to incorporate this feedback into their practice. Additionally, faculty and program leadership used aggregate information to identify knowledge and skills that could benefit from additional educational time at the group level. For example, residents frequently missed critical skills such as checking a finger-stick glucose in an unconscious patient, verbalizing a backup plan for intubation, specifying which blood products should be given in a trauma, and determining weight of a pediatric patient before giving medications. While some of these missed items could have been due to deficits in content knowledge, it is likely the cognitive load imposed on learners during these scenarios created room for errors. The complexity of task-switching places strain on learners' working memory, which can contribute to error.^21^ Assessments such as ours provide educators with an opportunity to help learners pinpoint those things that get missed in times of high cognitive load. However, further work is needed to demonstrate how this knowledge might mitigate errors in the clinical environment.

In implementing this curriculum, we learned several lessons. Although no content modifications were made throughout the day in order to ensure consistency, we did have to adjust some aspects of the scenarios to ensure everything ran as planned. For example, the outlet charging our manikin was located so that the manikin would come unplugged during intubations, necessitating that we reconfigure our room. We also reflected on several of the decisions we made in creating this curriculum. For instance, while return of spontaneous circulation from CPR alone is somewhat unusual, we chose not to include prehospital defibrillation in the case. We felt this choice successfully prompted the learner to think about postarrest care early in the case without providing too many diagnostic clues. Similarly, for feasibility reasons, we found it would be cumbersome to have multiple different arterial blood gases at different time points in scenario 1, as this could be very confusing, especially when using volunteers in the SP role. For this reason, one arterial blood gas that could represent the patient condition at various time points was used. We also reflected on the significant personnel required to run the simulation. While we were fortunate to have had many volunteers for this session, we understand that may not reflect the resources other programs have available. This course could be delivered with two or three fewer SPs, especially if an automated chest compression device is utilized. While not essential, we prefer using larger numbers of volunteers as it creates more realistic teams, which facilitates improved assessment and feedback around topics such as leadership and communication. To meet this need, we have recruited volunteers from a variety of sources, including nursing staff, medical students, department research assistants, retired paramedics, and the hospital volunteer program.

We also must note some important limitations to this work. First, the simulated environment was not as complex as the clinical environment of the ED. For example, residents were asked only to describe how to place a chest tube. Time constraints did not allow us to add simulated procedures. If faced with similar scenarios in the clinical environment, it is likely that participants would have demonstrated more challenges with completing all of the required tasks. We must also consider whether the simulation design itself contributed to some of the frequently missed items. For example, participants may not have asked for a family member because they assumed they had access only to what was in the room. While participants were briefed prior to the course, in the future it may help to provide them with a list of simulation environment capabilities and expectations prior to the session. In addition, while the checklists offered residents an opportunity to identify areas for improvement, our methods did not explore if they successfully incorporated these changes into their practice. The evaluation of our curriculum (Appendix O) was completed as a postsession survey with no presession comparison. This was done intentionally to avoid revealing the topics of the scenarios before the simulation; however, it limited our assessment of any change in confidence that may have occurred secondary to the simulation. Finally, we recognize that only nine of 12 participants completed the survey.

Future related work could include a delayed evaluation to assess how residents' reflections during the debrief impact how they manage patient care and task-switching in the clinical environment by employing our current checklist in an observational study of practice in the ED. Our team will also continue to refine our checklists, including adding a checklist item for postintubation sedation and a global checklist of task-switching skills, to augment the reflective debrief. Future research might also focus on how this type of simulation curriculum could be informed by techniques grounded in deliberate practice.^22^ Simulations can be designed to promote deliberate practice by emphasizing direct feedback and providing opportunities to reinforce skills.^23,24^ In fact, several participants in our study indicated that they would be interested in opportunities to complete the simulation again following the feedback they received. We agree that designing a curriculum that allows learners to reinforce their skills is an effective way to promote lifelong learning. One possible way to incorporate deliberate practice might be to provide an additional opportunity to participate in a simulation involving similar skills closer to graduation. Overall, this simulation was received well by learners, who felt it promoted reflection on their skills in clinical care, leadership, and task-switching that they could apply to their practice. This simulation provides a tool for educators to address the challenge of assessing core competencies, including clinical resuscitation skills, leadership, and task-switching.

## Appendices


Scenario 1.docxScenario 1 Setup and Prompts.docxScenario 1 Stimuli.pptxScenario 1 Skills Checklist.docxScenario 2.docxScenario 2 Setup and Prompts.docxScenario 2 Adult Stimuli.pptxScenario 2 Peds Stimuli.pptxScenario 2 Skills Checklist.docxScenario 3.docxScenario 3 Setup and Prompts.docxScenario 3 Skills Checklist.docxExample Schedule.xlsxDebriefing Material.docxPostsession Evaluation.docx
*All appendices are peer reviewed as integral parts of the Original Publication.*
