# Supplementary material for: Capstone Simulation: A Multipatient Simulation for Senior Emergency Medicine Residents
Source: MedEdPORTAL. 2023 Nov 9;19:11361. doi: 10.15766/mep_2374-8265.11361 (PMC10632183; doi:10.15766/mep_2374-8265.11361)
Supplement: Supplementary file 1 — Scenario 1.docxScenario 1 Setup and Prompts.docxScenario 1 Stimuli.pptxScenario 1 Skills Checklist.docxScenario 2.docxScenario 2 Setup and Prompts.docxScenario 2 Adult Stimuli.pptxScenario 2 Peds Stimuli.pptxScenario 2 Skills Checklist.docxScenario 3.docxScenario 3 Setup and Prompts.docxScenario 3 Skills Checklist.docxExample Schedule.xlsxDebriefing Material.docxPostsession Evaluation.docx [file mep_2374-8265.11361-s001.zip › I. Scenario 2 Skills Checklist.docx]

**Appendix I: Scenario 2 Skills Checklist**

*Unless clearly stated otherwise, please DO NOT give credit even for prompted answers. However, you should take note of which items required prompting to help facilitate the debrief.*

Resident Name: __________________ Date: ______________

Adult Patient – initial management:

Primary survey:

- - Examines airway (e.g., verbalizes ETT depth, looks in mouth)
  - Confirms ETT placement (e.g., ETCO2, DL)
  - Chest auscultation
  - Pulse check
- Confirms existing IV access.
- Performs secondary survey.
- Rolls patient.

Pediatric Patient – initial management:

Primary Survey:

- - Examines airway.
  - Chest auscultation
  - Pulse check
- Requests dosing aid (e.g., Broselow tape) or weight
- Requests monitor
- Puts patient in c-collar OR verbalizes rationale for not doing it.
- Performs secondary survey.
  - Performs pulse check in right lower extremity.
- Rolls patient.

Adult Patient – resuscitation:

- Reassesses patient after initial evaluation of pediatric patient.

(e.g., repeats exam or asks RN for updated exam or vital signs)

Verbalizes vital sign abnormalities.

- - Hypoxemia ☐ Hypotension
- Recognizes pneumothorax within 10 min of arriving (prior to RN or Trauma Surgeon prompting)
- Verbalizes intent to perform tube or needle thoracostomy (will place a “verbal chest tube”)
- Orders CXR (prior to RN prompting, ok if after chest tube placement)
- Orders Pelvis XR
- Orders FAST (prior to RN prompting)
- Orders a Type and Cross or Type and Screen
- Orders 2^nd^ PIV
- Recognizes free fluid on FAST.
- Initiates resuscitation with blood products (Prior to RN or Trauma Surgeon prompting, must specify transfusion, does not count if ordered to have it “available” or “at bedside”)
  - Type O (+ or -) uncrossmatched (okay if RN asks…. “What type of blood do you want?”)
  - Specifies 1:1 ratio
- Orders TXA (tranexamic acid)
- Requests trauma surgeon
- No vasopressors given or, if vasopressors are ordered/discussed, a rationale provided (e.g., concern for neurogenic shock): _____________________________________________________________________

Pediatric Patient – management:

- Reassesses patient (e.g., examines or rechecks VS at least once after initial assessment)
- Correctly dose pain medication by using weight (~20 kg) or appropriate color on dosing guide (e.g., blue on Broselow). OK if prompted by a nurse to order something. Some viable options:

Ibuprofen 10 mg/kg (200 mg)

APAP 10-15 mg/kg (200-300 mg)

Ketorolac 0.5 mg/kg (10 mg)

Morphine 0.05-0.1 mg/kg (1-2 mg)

Hydrocodone 0.1-0.15 mg/kg (2-3 mg)

Oxycodone 0.05-0.15 mg/kg (1-3 mg)

- Does not obtain unnecessary imaging (e.g., “pan-scan”). Comments: _____________

_____________________________________________________________________

- Verbalizes plan to splint RLE

Team Leadership:

- Assigns roles/delegates tasks.
- Provides at least one update/”huddle” to the team.
- Encouraged input from the team at least once.

(e.g., “Any questions?,” “ If you have questions or concerns, please speak up.”)

Task-switching

- Identifies priorities between multiple patients.

(e.g., completes sufficient assessment of pediatric patient to determine patient is lower acuity)

- Appropriately allocates resources between two patients.

(e.g., most staff for adult patient but ensures at least one person is reassessing peds patient)

- Prioritizes urgent/emergent issues.

(e.g., delegates or postpones low acuity issues on peds patient to avoid delays for adult patient)

- Facilitates simultaneous management of two patients.

(e.g., creates plan for patient to enable team to move forward with care while leader absent)

Other Comments: _______________________________________________________________________

__________________________________________________________________
