# Supplementary material for: Capstone Simulation: A Multipatient Simulation for Senior Emergency Medicine Residents
Source: MedEdPORTAL. 2023 Nov 9;19:11361. doi: 10.15766/mep_2374-8265.11361 (PMC10632183; doi:10.15766/mep_2374-8265.11361)
Supplement: Supplementary file 1 — Scenario 1.docxScenario 1 Setup and Prompts.docxScenario 1 Stimuli.pptxScenario 1 Skills Checklist.docxScenario 2.docxScenario 2 Setup and Prompts.docxScenario 2 Adult Stimuli.pptxScenario 2 Peds Stimuli.pptxScenario 2 Skills Checklist.docxScenario 3.docxScenario 3 Setup and Prompts.docxScenario 3 Skills Checklist.docxExample Schedule.xlsxDebriefing Material.docxPostsession Evaluation.docx [file mep_2374-8265.11361-s001.zip › L. Scenario 3 Skills Checklist.docx]

**Appendix L: Scenario 3 Check-list**

*Unless clearly stated otherwise, please DO NOT give credit for prompted answers. However, you should take note of which items required prompting to help facilitate the debrief.*

Resident Name: _________________________ Date: ___________

Introduction:

- Introduces self and role
- Confirms family member’s relationship to the patient
- Inquires what the family member already knows
- Gives a warning – (e.g., “I have some bad news for you….”)
- Avoids medical jargon
- Avoids euphemisms
- Gives important news without unnecessary delay

Interpersonal skills:

- Appropriate affect
- Acknowledges family member’s feelings

Transitioning:

- Inquires if the family member has any questions
- Informs family member of the next step (e.g., seeing the patient, waiting in family room, etc.)
- Offers support services (e.g., social worker, chaplain, calling additional family, etc.)

Comments:___________________________________________________________________________________________________________________________________________________________________________________________________________________________________________________________________________________________________________________________________________
