# Supplementary material for: Capstone Simulation: A Multipatient Simulation for Senior Emergency Medicine Residents
Source: MedEdPORTAL. 2023 Nov 9;19:11361. doi: 10.15766/mep_2374-8265.11361 (PMC10632183; doi:10.15766/mep_2374-8265.11361)
Supplement: Supplementary file 1 — Scenario 1.docxScenario 1 Setup and Prompts.docxScenario 1 Stimuli.pptxScenario 1 Skills Checklist.docxScenario 2.docxScenario 2 Setup and Prompts.docxScenario 2 Adult Stimuli.pptxScenario 2 Peds Stimuli.pptxScenario 2 Skills Checklist.docxScenario 3.docxScenario 3 Setup and Prompts.docxScenario 3 Skills Checklist.docxExample Schedule.xlsxDebriefing Material.docxPostsession Evaluation.docx [file mep_2374-8265.11361-s001.zip › N. Debriefing Material.docx]

**Simulation Capstone Assessment Debriefing Materials (Appendix N)**

Debriefing Instructions:

1. Conduct in a quiet and private space to facilitate an uninterrupted debrief.
2. We suggest using the Gather-Analyze-Summarize (GAS) method of debriefing:
   1. Gather: Actively listen to the participant and ask for their self-reflection on the suggested questions.
   2. Analyze: Provide your feedback and build on their self-reflection as appropriate, but also be sure to refute misperceptions when appropriate. These misperceptions can be in either direction, with resident learners frequently being overly self-critical or under-recognizing their strengths. Use the checklist to provide specific examples that support the participant’s strengths and areas for improvement. Review the clinical portion of the checklist for any missed items not already addressed through previous steps.
   3. Summarize: End the debrief by asking the participant to summarize 2-3 important concepts they want to integrate into their care.

Scenario 1:

1. What did you do well when managing the ACLS scenario?
2. What could you have done better in managing the ACLS scenario?
3. What did you do well to manage the airway of the patient in the ACLS scenario?
4. What could you have done better in managing the airway of the patient in the ACLS scenario?

Scenario 2:

1. What did you do well in managing the adult ATLS scenario?
2. What could you have done better in managing the adult ATLS scenario?
3. What did you do well in managing the pediatric ATLS scenario?
4. What could you have done better in managing the pediatric ATLS scenario?

Scenario 3:

1. How did it feel to deliver bad news to the patient’s family member?
2. What did you do well during that conversation?
3. What could you have done better during that conversation?

Task-switching:

1. How did it feel to task-switch between scenarios and between patients within a scenario?
2. What, if anything, made it difficult?
3. What strengths do you think you have when task-switching?
4. What could you have done better in task-switching between patients and between scenarios?

**Additional helpful tips for the debrief:**

Keys areas of effectiveness observed:

- Active listening facilitates learners centered debriefs.
- Eliciting participants emotional states allows learners to be vulnerable in their reflections.
- While responding to the participant’s reflections is important, it is also helpful to report your own observations to ensure all key points are discussed.
- At times learners may get off track. It is helpful to refocus on them with phrases such as: “I understand, however, tell me about “X” aspect of the scenario…”
- Asking learners to describe what they want to incorporate into their practice is a useful way to summarize those things they took away from the reflection.

Keys areas of effectiveness observed:

- Providing faculty observations first hinders learners’ ability to reflect and provide rich insights on their own strengths and weaknesses.
- Spending too much time on all the missed items of the check-list hinders learner reflection and takes too much of the allotted time.
- It is important to keep an eye on the time of the debrief to ensure there is time for the learner to focus on those things they will take away from the simulation.

***An example dialogue (Scenario 3):***

Faculty Observer: “Tell me how it felt to deliver bad news to the patient’s family member?”

Resident: “At first, it was a little uncomfortable. I wasn’t sure what the family member knew already, and I fumbled a little when I delivered the initial news. That made me anxious, and it took me a little while to re-establish report with the family member and ensure he had all the information I needed. I was worried he could tell I was anxious.”

Faculty Observer: “I agree that you fumbled a little at first but were able to connect with the family member by the end of the conversation. I don’t think you came across as anxious during the conversation. We can talk about what you might do differently next time, but first, tell me what you think you did well during that conversation.”

Resident: “I used empathetic language and appropriate body language. I also gave the family member some time to absorb what they had heard by pausing and allowing for silence. This was how I was able to connect with him. I felt like I had answered all his questions by the end of our conversation.”

Faculty Observer: “I agree. You leaned in when talking to the patient and made good eye contact with him. You were also able to slow down to ensure he understood everything you were saying to him, which is extremely important when you must deliver shocking news to someone. Now, you mentioned that the conversation started off a little uncomfortable. What do you think you could have done better during the conversation?

Resident: “I should have started by asking the family member what he knew about his family members status already. That way I wouldn’t have had to back-track when the family member couldn’t understand what happened prior to his arrest. I should also have used the work “died” instead of “passed away” when delivering bad news.”

Faculty Observer: “Excellent reflection. It is important to avoid euphemisms when delivering bad news. Those were the same things I noted you missed on the check-list. As you can see you successfully completed all the other tasks.”

Then after all scenario debriefs are completed:

Faculty Observer: “Summarize for me 2-3 important things you will take away from this exercise to incorporate into your care.”

Resident: “I will…1) ensure that I pre-charge the defibrillator when running a code, 2) start conversations where I am breaking bad news by asking the family member what they understand about their family member’s status thus far, and 3) give team members more summary statements so that they can help me care for patients when I need to task-switch between patients.”
