# Supplementary material for: Capstone Simulation: A Multipatient Simulation for Senior Emergency Medicine Residents
Source: MedEdPORTAL. 2023 Nov 9;19:11361. doi: 10.15766/mep_2374-8265.11361 (PMC10632183; doi:10.15766/mep_2374-8265.11361)
Supplement: Supplementary file 1 — Scenario 1.docxScenario 1 Setup and Prompts.docxScenario 1 Stimuli.pptxScenario 1 Skills Checklist.docxScenario 2.docxScenario 2 Setup and Prompts.docxScenario 2 Adult Stimuli.pptxScenario 2 Peds Stimuli.pptxScenario 2 Skills Checklist.docxScenario 3.docxScenario 3 Setup and Prompts.docxScenario 3 Skills Checklist.docxExample Schedule.xlsxDebriefing Material.docxPostsession Evaluation.docx [file mep_2374-8265.11361-s001.zip › O. Postsession Evaluation.docx]

Dear Resident,

Thank you for completing the PGY3 Capstone Simulation. We would appreciate any feedback you can provide to us about your experience. This feedback will also allow us to share this educational exercise with others. No identifiable information will be collected and participation in this survey is voluntary. Please feel free to reach out to us with any questions. **Here is a link to an anonymous**  **[survey to be uploaded online]**

*Please rate your agreement with the following statements:*

|  |  | Strongly Disagree | Disagree | Neither Agree nor Disagree | Agree | Strongly Agree |
| --- | --- | --- | --- | --- | --- | --- |
| 1. | This simulation case provided is relative to my work. | □ | □ | □ | □ | □ |
| 2. | This simulation case was realistic. | □ | □ | □ | □ | □ |
| 3. | The debrief was a safe environment. | □ | □ | □ | □ | □ |
| 4. | The debrief promoted reflection. | □ | □ | □ | □ | □ |
| 5. | This simulation case was effective in promoting feedback for areas to focus on prior to graduation. | □ | □ | □ | □ | □ |

**After participating in this session, how confident are you in your ability to:**

|  |  | Very Not confident | Not confident | Neutral | Confident | Very Confident |
| --- | --- | --- | --- | --- | --- | --- |
| 6. | Manage a patient in cardiac arrest. | □ | □ | □ | □ | □ |
| 7. | Evaluate a patient after traumatic injury. | □ | □ | □ | □ | □ |
| 8. | Manage multiple patients simultaneously. | □ | □ | □ | □ | □ |
| 9. | Deliver bad news to a patient family member. | □ | □ | □ | □ | □ |
| 10. | Task switch between patients of different acuity levels. | □ | □ | □ | □ | □ |
| 11. | Self-reflect on my care and identify areas for improvement. | □ | □ | □ | □ | □ |

12. What did you take away from this case and/or how will it change your practice?

____________________________________________________________________

____________________________________________________________________

____________________________________________________________________

13. What specific changes would you make to improve this scenario?

____________________________________________________________________

____________________________________________________________________

14. Other comments or suggestions:

____________________________________________________________________

____________________________________________________________________

____________________________________________________________________

**Thank you for taking the time to complete this survey!**
