# Supplementary material for: Capstone Simulation: A Multipatient Simulation for Senior Emergency Medicine Residents
Source: MedEdPORTAL. 2023 Nov 9;19:11361. doi: 10.15766/mep_2374-8265.11361 (PMC10632183; doi:10.15766/mep_2374-8265.11361)
Supplement: Supplementary file 1 — Scenario 1.docxScenario 1 Setup and Prompts.docxScenario 1 Stimuli.pptxScenario 1 Skills Checklist.docxScenario 2.docxScenario 2 Setup and Prompts.docxScenario 2 Adult Stimuli.pptxScenario 2 Peds Stimuli.pptxScenario 2 Skills Checklist.docxScenario 3.docxScenario 3 Setup and Prompts.docxScenario 3 Skills Checklist.docxExample Schedule.xlsxDebriefing Material.docxPostsession Evaluation.docx [file mep_2374-8265.11361-s001.zip › D. Scenario 1 Skills Checklist.docx]

**Appendix D: Scenario 1 Checklist – Airway Management and ACLS**

*Unless clearly stated otherwise, please DO NOT give credit for prompted answers. However, you should take note of which items required prompting to help facilitate the debrief.*

Resident Name: ____________________ Date: __________

Airway Management:

Initial Management:

- Requests/confirms IV access (patient has two 18 ga PIVs)
- Requests blood sugar
- Performs basic physical exam.
- Recognizes hypoxemia prior to prompt from RN (approx 2 min) by verbalization or taking corrective measures (e.g., initiating BVM, turning up oxygen)
- Verbalizes intent to intubate prior to RN prompt (approx 5 min)
- Ask for family (or social work to help find family) – can happen any time during the case.

Preparation:

- Inspects airway (e.g., looks in mouth and/or assesses 3-3-2 rule)
- Preoxygenation – BVM or NRB while gathering supplies.
- Oxygen at >10 L/min
- Confirms laryngoscope light is functioning.
- Confirms suction is working.
- Positions bed/patient
- Evaluates or discusses hemodynamics (e.g., blood pressure)
- Verbalizes plan for intubation.
- Verbalizes back-up plan for intubation.

Medications (patient ~70kg):

- Sedative:
- Etomidate □ Correct dose - 0.3 mg/kg (~20 mg)
- Propofol □ Correct dose - 1.5-3 mg/kg (~100-200 mg)
- Ketamine □ Correct dose - 1.5-2 mg/kg (~100- 150 mg)
- Other: _______________
- Paralytic:
  - Succinylcholine □ Correct dose - 1.5-2 mg/kg (~100-150 mg) or
  - Rocuronium □ Correct dose - 1-1.5 mg/kg (~70-100 mg)

If No RSI:

- - Rationale provided. Comments:____________________________________

Intubation:

- Completes intubation on first attempt. If not, note number of attempts: _________
- Inflates cuff.

Confirms Tube Placement:

- Verbalizes visualization of the cords
- Requests ETCO2 detector and/or monitoring
- Auscultation (performs or delegates team member)
- Orders CXR

Post-intubation Management:

- Secures ETT (or delegates team member to do it)
- Verbalizes sedation medications or provides rationale for not sedating.
- Orders ventilator settings.
- Orders foley catheter
- Orders OG/NG tube placement
- Elevates head of bed
- Orders ABG

STEMI Management:

- Correct ECG interpretation (e.g., STEMI, anterior infarct, MI)
- Orders placement of pads for defibrillator
- Orders aspirin
- Activates cath lab (e.g., calling a “code STEMI,” requesting cath lab activation, etc. ) - do not give credit if RN must prompt.

Cardiac Arrest:

Recognition and Initial Management:

- Recognizes cardiac arrest prior to RN prompt – either by verbalizing vfib or lack of pulses
- Verbalizes correct rhythm identification within the first cycle of CPR.
- Initiates chest compressions within 10 seconds of recognizing cardiac arrest.
- Requests defibrillator within 20 seconds of recognizing cardiac arrest.
- Checks central pulses (or delegated team member)

CPR and Defibrillation:

- Verbalizes target bagging rate and/or corrects team member performance.
- Verbalizes target compression rate and/or corrects team member performance.
- Requests ETCO2 monitoring
- Defibrillates immediately (e.g., as soon as set-up, does not wait for 2-minute mark)
- Defibrillates with correct energy (200 or 360J)
- Defibrillates at the 2-minute interval.
- Charges for defibrillation prior to pulse check
- Coordinates pulse/rhythm checks, defibrillations, etc.
- Resumes chest compressions immediately following defibrillation.
- Does not interrupt compressions for other diagnostics (e.g., ECG, CXR)

Pharmacotherapy:

- Orders Epinephrine
  - Orders correct dose of Epinephrine 1mg.
- Orders anti-arrhythmic
  - Orders correct dose of anti-arrhythmic:
  - Amiodarone 300mg IV push OR
  - Lidocaine 1.5 mg/kg (~100 mg)

Post-ROSC Management:

- Requests ECG - do not give credit if RN provides ECG without an order.
- Requests CXR
- Requests blood pressure
- Discusses or initiates therapeutic hypothermia.

Team Leadership:

- Assigns roles/delegates tasks.
- Provides at least one update/”huddle” to the team.
- Encouraged input from the team at least once.

(e.g., Any questions? If you have questions or concerns, please speak up)

Other comments: __________________________________________________________________________________________________________________________________________________________________________
