# Supplementary material for: Capstone Simulation: A Multipatient Simulation for Senior Emergency Medicine Residents
Source: MedEdPORTAL. 2023 Nov 9;19:11361. doi: 10.15766/mep_2374-8265.11361 (PMC10632183; doi:10.15766/mep_2374-8265.11361)
Supplement: Supplementary file 1 — Scenario 1.docxScenario 1 Setup and Prompts.docxScenario 1 Stimuli.pptxScenario 1 Skills Checklist.docxScenario 2.docxScenario 2 Setup and Prompts.docxScenario 2 Adult Stimuli.pptxScenario 2 Peds Stimuli.pptxScenario 2 Skills Checklist.docxScenario 3.docxScenario 3 Setup and Prompts.docxScenario 3 Skills Checklist.docxExample Schedule.xlsxDebriefing Material.docxPostsession Evaluation.docx [file mep_2374-8265.11361-s001.zip › A. Scenario 1.docx]

| **Appendix A: *Scenario 1***  **SIMULATION CASE TITLE: Capstone Case 1: ACLS, airway and STEMI Management**  **AUTHORS: Caitlin Schrepel, MD, Anne Chipman, MD, MS, Ross Kessler, MD, Crystal Phares, MD, Elizabeth Rosenman, MD**  **LEARNER AUDIENCE: PGY3 or PGY4 Emergency Medicine Residents** | |
| --- | --- |
| **PATIENT NAME: Thomas**  **PATIENT AGE: 43 years old**  **CHIEF COMPLAINT: Brought in by EMS after a witnessed collapse and bystander CPR.**  **PHYSICAL SETTING: Emergency department resuscitation bay** | |
|  | |
| **Brief narrative description of case** | *The learner is informed by paramedics that they brought in a 43-year-old male patient who had witnessed a collapse while walking to work and received bystander CPR. The patient arrives with pulses intact and is placed on the monitor. He becomes hypoxemic, necessitating intubation. ECG demonstrates an ST-elevation myocardial infarction necessitating activation of the cardiac cath lab and medical management. Following intubation and confirmation of tube placement, the patient loses pulses requiring ACLS management for a ventricular fibrillation arrest. The patient will regain pulses after three to four rounds of CPR, with defibrillation. The learner must then begin post-arrest evaluation, until the patient is transported for cardiac catheterization.* |
| **Primary Learning Objectives** | *By the end of this session, learners will be able to:*  *Demonstrate the ability to lead a team while managing an acutely ill patient.*  *Demonstrate appropriate airway management.*  *Apply ACLS guidelines appropriately for a patient in ventricular fibrillation.*  *Recognize and manage STEMI appropriately.* |
| **Critical Actions** | 1. *Assess airway, breathing and circulation upon patient arrival.* 2. *Recognize hypoxemia and stabilize with oxygen and airway adjuncts.* 3. *Intubate the persistently hypoxemic patient and confirm airway placement.* 4. *Recognize the patient's loss of pulses and identify rhythm as ventricular fibrillation.* 5. *Manage ventricular fibrillation arrest, including use of CPR, defibrillation, and appropriate medications.* 6. *Demonstrate leadership while running a code by assigning roles and using closed loop communication.* 7. *Recognize STEMI on ECG and activating the cardiac catheterization lab* |
| **Learner Preparation or Prework** | *Learners were briefed on the following before Scenario 1:*  Environment: Community hospital  ---Community hospital with consultants available by phone.  ---OR, acute care, and ICU admissions available.  Team: Will be in the room, but you can ask for more resources as needed.  Simulation: Reminder of manikin capabilities. Reminder to ask the RN if there are any questions about fidelity or availability of resources.  Questions: Any resident questions were clarified. |

| **Initial Presentation** | | | |
| --- | --- | --- | --- |
| **Initial vital signs** | *BP 145/90, HR 110, O2 Sat 92% on 2L of oxygen by nasal cannula, RR 8* | | |
| **Overall Setting and Appearance** | *When the learners enter the room they are in a simulated emergency department resuscitation bay. The patient is on the stretcher in a gown with two peripheral IVs placed. The code cart, airway cart, and IV polls are in the room just as they would be in a resuscitation room. The nurse and paramedic are at the bedside on participant arrival.* | | |
| **Standardized Participants (and their roles in the room at case start**) | *As the participant enters the room the paramedic will provide the initial history. The paramedic states, “This is Thomas. He is a 43-year-old male who had a witnessed collapse and bystander CPR. He was walking with work colleagues when he collapsed. His coworkers did CPR for about 5 min. He had pulses when EMS arrived. We have established two 18 gauges in his bilateral ACs. According to his coworkers, he hadn’t been complaining about anything and didn’t appear sick. He moans to sternal rub, but that’s it. This occurred 2 blocks away, so we decided just to bring him here without doing too much. Past medical history, meds, allergies, are all unknown. HR 110, BP 145/90, O2 sat 92% on 2L.”*  *The paramedic should stay in the room to offer help. In addition to the bedside nurse, a medical assistant and respiratory therapist are available outside the room should the learner ask for additional help.* | | |
| **HPI** | *Patient moans but cannot provide additional history. There is no other history available from other sources.* | | |
| **Past Medical/Surgical History** | **Medications** | **Allergies** | **Family History** |
| *Unknown* | *Unknown* | *Unknown* | *Unknown* |
| **Physical Examination** | | | |
| **General** | *Somnolent, eyes closed, minimally responsive (slight groan to deep sternal rub).* | | |
| **HEENT** | *Atraumatic, pupils equal and reactive* | | |
| **Neck** | *No C spine step offs, no masses* | | |
| **Lungs** | *Decreased respiratory rate, bilateral breath sounds* | | |
| **Cardiovascular** | *Regular rate and rhythm, no murmurs* | | |
| **Abdomen** | *Soft, non-distended* | | |
| **Neurological** | *Minimally responsive, GCS 7, moans to deep sternal rub, no eye opening, slight withdrawal to pain (verbalized by bedside RN if learner tests this)* | | |
| **Skin** | *No lacerations, bruises, or rashes* | | |
| **GU** | *Normal* | | |
| **Psychiatric** | *N/A* | | |

| **Instructor Notes - Changes and CASE Branch Points**  ***See Appendix B for additional instructions and prompt handouts for this case.*** |
| --- |

**
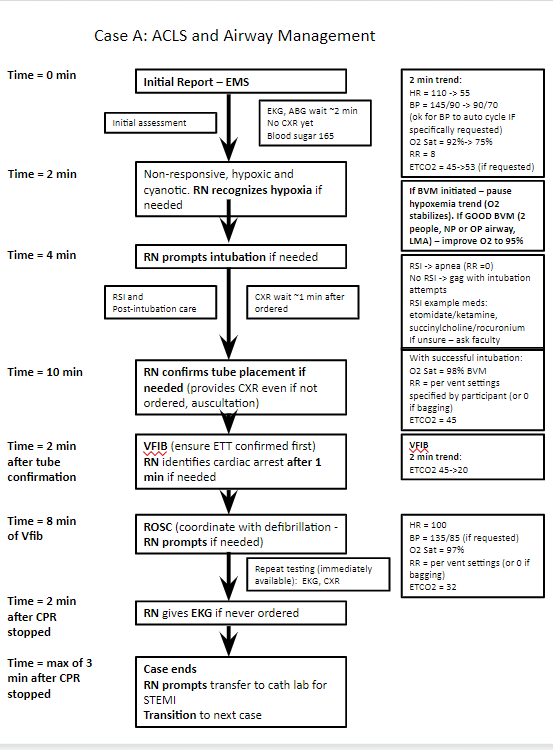
**

**Citation: Image is Author Created and Owned**

| **Instructor Notes - Changes and CASE Branch Points** | | |
| --- | --- | --- |
| **Time Point** | **Change in Case/Actions** | **Additional Information** |
| *Time 0 (Start of Case)* | EMS provides history  Blood glucose available if requested  ABG or EKG available ~2 min after ordered  CXR not available until after intubation | This is Thomas. He is a 43 yo Male who had a witnessed collapse and bystander CPR. He was walking with work colleagues when he collapsed. His coworkers did CPR for ~5 min. He had pulses when EMS arrived. We have two 18 gauges in his bilateral ACs. According to his coworkers, he hadn’t been complaining about anything and didn’t appear sick. He moans to sternal rub, but that’s it. This occurred 2 blocks away, so we decided just to bring him here without doing too much. Past medical history, meds, allergies, are all unknown. HR 110, BP 145/90, O2 sat 92% on 2L.  Do not provide glucose unless specifically asked. |
| *Time 0 - 2 mins* | Pt becomes more hypoxemic, bradycardic and hypotensive as noted in the 2 min trend on the right of the flow chart above. (This occurs regardless of participant actions) | EtCO2 should be added to trend only if asked specifically.  If hypoxemia is not recognized by 2 minutes the RN should prompt by stating: “Did you see his saturation doctor?” |
| *Time 2-4 minutes* | Pt is unresponsive.  Participant should work to improve SpO2 and verbalize plan for intubation. | If good BVM is performed at any time, then SpO2 should improve to 95% and other vitals stabilize.  NRB or other oxygen support will not improve SpO2 as pt is unresponsive.  RN prompts intubation if plan not verbalized by 4 minutes : “He doesn’t seem to be getting much better with bagging, should we go ahead and intubate?” |
| *Time 4-10 minutes* | Pt is intubated and post-intubation care is started.  CXR available 1 minute after intubation.  Vital signs improve following intubation as noted on the right of the flowchart above. | If the learner is unable to intubate, then paramedic intubates (e.g., multiple failed attempts): “I’m certified to intubate; do you mind if I take one look?”  If the learner requests anesthesia back-up the RN can page anesthesia but then inform the learner that anesthesia is not available.  If the learner requests a ventilator, RN asks the learner to specify ventilator settings. |
| *Time 11 minutes* | RN provides CXR and auscultates lungs to confirm ETT placement if not already done. The goal is to confirm ETT placement prior to cardiac arrest so the learner doesn’t extubate or perform needle decompression. |  |
| *Time 12 minutes or 2 min after tube placement confirmed* | V-fib arrest occurs (this will happen regardless of any learner actions)  EtCO2 trend during Vfib begins as noted on the flow chart above | If Vib is not identified after 1 minute, RN identifies cardiac arrest if needed: “I can’t feel a pulse.” |
| *Time 12 minutes -20 minutes* | ~8 minutes of total V-fib (this will occur regardless of any learner actions) | If learner does not identify Vfib, RN should prompt:  “Do you think we should try defibrillating?”  No prompts for medication are given. |
| *Time 20 minutes* | ROSC is achieved  Vital signs improved as per box on the right of the flow chart above. |  |
| *Time 20 - 23 minutes* | Post-arrest care  EKG and CXR available if requested.  Management of STEMI with activation of cath lab | If not requested by 2 minutes after ROSC, RN provides the learner with post-arrest ECG.  If a learner orders an ECG, provide it immediately after chest compressions are stopped.  RN wraps up the case at ~3 after ROSC:  “The cath lab just called. They are ready for the patient. Is there anything else you want before we go?” |

**Ideal Scenario Flow**

The scenario will start with a report from EMS about an adult patient who had bystander CPR after collapsing. On arrival the patient will have pulses with relatively normal vital signs (HR 110, BP 145/90, O2 sat 92% on 2L), but responding only to noxious stimuli. The participant will start an evaluation of the patient. During the first 2 minutes the patient will become more hypoxemic, bradycardic, and hypotensive. The participant will then need to correct hypoxemia by assisting respirations with BVM. Once hypoxia improves, the other vital signs will improve and stabilize. However, the patient will remain unresponsive and require intubation. The patient should be intubated with RSI. After intubation, the placement of the ETT will be confirmed with exam, EtCO2, and CXR. Regardless of participant actions, 2 minutes after intubation the patient will lose pulses requiring ACLS management for a ventricular fibrillation arrest. The participant will lead the team in the management of a Vfib arrest with the use of defibrillation, compressions, and medical management. The patient will regain pulses with defibrillation after ~3 cycles of CPR. After ROSC is achieved, the participant will start post-arrest management. An electrocardiogram will then demonstrate a STEMI and the participant should verbalize the plan for activation of the cardiac catheterization lab and medical management. The case will then end with the charge nurse arriving to tell the participant about their next patient.

***As this patient was getting prepared to transfer to the cath lab, a faculty observer entered the room to inform the resident that they had another patient.***

**Anticipated Management Mistakes**

1. Staying on-time with 3 rounds of CPR: You may decide to condense the cycles of CPR to be a little shorter (~1.5 minutes). Don’t tell the participant you are doing this (they should still think it is 2 minutes. The person asked to keep time should say: “it’s been two minutes.” The decision to do this can be made based on scheduling factors and the team’s capacity for performing compressions.

2. Failure to recognize the need for intubation: Some participants may try to delay intubation by using a non-rebreather or nasal cannula. Given that the patient is unresponsive and not breathing well on their own, this will not be sufficient. We have found it helpful to worsen hypercarbia and hypoxemia if this occurs.

3. Failure to remember to check a point of care glucose in an unresponsive patient: We found that many of our learners forgot this important step in assessment. This was highlighted during debriefs as targeted feedback.

4. Failure to defibrillate: While this was not a problem we encountered; this may be a more frequent occurrence if the case is used with more junior learners. We coordinate ROSC with a defibrillation and would recommend a back-up prompt (e.g., a suggestion from the SP nurse) to force defibrillation if the learner does not make this decision after 3 cycles of CPR.
