# Supplementary material for: Capstone Simulation: A Multipatient Simulation for Senior Emergency Medicine Residents
Source: MedEdPORTAL. 2023 Nov 9;19:11361. doi: 10.15766/mep_2374-8265.11361 (PMC10632183; doi:10.15766/mep_2374-8265.11361)
Supplement: Supplementary file 1 — Scenario 1.docxScenario 1 Setup and Prompts.docxScenario 1 Stimuli.pptxScenario 1 Skills Checklist.docxScenario 2.docxScenario 2 Setup and Prompts.docxScenario 2 Adult Stimuli.pptxScenario 2 Peds Stimuli.pptxScenario 2 Skills Checklist.docxScenario 3.docxScenario 3 Setup and Prompts.docxScenario 3 Skills Checklist.docxExample Schedule.xlsxDebriefing Material.docxPostsession Evaluation.docx [file mep_2374-8265.11361-s001.zip › B. Scenario 1 Setup and Prompts.docx]

**Appendix B: Scenario 1 Set-up**

Mannequin Set-up:

- High-technology human patient simulator
- Wearing patient gown
- Bed at lowest position
- Two 18 ga IVs in place
- 1L NS or LR hanging.
- 2L NC on patient
- Monitor:
  - Telemetry, BP cuff, pulse oximetry at bedside but NOT hooked up.
  - Monitor off.
  - Initial VS: HR 110 in sinus rhythm, BP 145/90, RR 12, 92% on 2 L NC
  - Positioned within camera view.

Additional Equipment:

- Ventilator near the head of bed
- Code cart with airway supplies
- Positioned of to the side
- LifePak and pads
  - Second set of pads
  - Various size blades and ETTs
  - Bougie/Eschmann stylet
  - LMA
  - OP and NP airways, tongue blades
  - Ambu bag and mask
  - Nasal cannula, venti mask, non-rebreather mask
  - Suction
  - 1 opened ETCO2 detector
  - Tape for ETT ties for securing ETT and OG/NG tube
  - Cricothyroidotomy kit – percutaneous or open ok
  - Penlight
- Empty mayo stand
- Blankets and/or sheets for stacking
- Glidescope
- Foot stool for CPR
- Computer for images
- Telephone for calling/receiving calls from the control room

AV Needs:

- Lapel microphone for the learner
- Radios with earpiece for SP RN
- Radio without earpiece for communicating from control room to SP RN

*Prompt times are approximate and should be used as guidelines to keep the case and participant on track.*

Primary roles: RN1 and Paramedic

Additional roles: 1 MA, 1 RT (outside room, and can enter if a learner requests help)

Sim Technician

Faculty Instructor

The paramedic and RN are in the room at the start of the case. The paramedic should remain in the room after the report to offer help. Additional personnel are available if the learner requests additional help.

**Time = 0 min**

*Paramedic provides history.*

This is Thomas. He is a 43 yo Male who had a witnessed collapse and bystander CPR. He was walking with work colleagues when he collapsed. His coworkers did CPR for ~5 min. He had pulses when EMS arrived. We have two 18 gauges in his bilateral ACs. According to his coworkers, *he hadn’t been complaining about anything and didn’t appear sick*. He moans to sternal rub, but that’s it. This occurred 2 blocks away, so we decided just to bring him here without doing too much. Past medical history, meds, allergies, are all unknown. HR 110, BP 145/90, O2 sat 92% on 2L.

*Do not provide glucose unless specifically asked.*

**Time = 2 min**

*RN recognizes hypoxemia if not already noted by the learner:*

“Did you see his saturation doctor?”

**Time = 4 min**

*RN prompts intubation if needed:*

“He doesn’t seem to be getting much better with bagging, should we go ahead and intubate?”

**Time = 8-10 min**

If the learner is unable to intubate, then *paramedic intubates (e.g., multiple failed attempts):*

“I’m certified to intubate; do you mind if I take one look?”

*If the learner requests anesthesia back-up the RN can page anesthesia but then inform the learner that anesthesia is not available.*

**Post-intubation**

If the learner requests ventilator, *RN asks the learner to specify ventilator settings.*

*RN provides CXR* ~1min after ordered.

**Time = 11 min**

RN provides CXR and auscultates lungs to confirm ETT placement if not already done. The goal is to confirm ETT placement prior to cardiac arrest so the learner doesn’t extubate or perform needle decompression.

**Time = 12 minutes** or 2 min after tube placement confirmed

Vfib arrest occurs..

**Time = 13 minutes** or 1 min after Vfib arrest

*RN identifies cardiac arrest if needed:*

“I can’t feel a pulse.”

**Time = ~6-8 min after Vfib arrest (start at time of arrest NOT at time learner recognized arrest) – this should equal 3-4 cycles of CPR**

ROSC – sinus rhythm

This is approximate and should be timed with a defibrillation (e.g., ok to do at 7 min if defibrillation occurs then)– *RN should prompt final defibrillation if needed:*

“Do you think we should try defibrillating?” or “It’s been two minutes, should we try defibrillating again?”

** You may decide to condense the cycles of CPR to be a little shorter (~1.5 minutes). Don’t tell the participant we are doing this (they should still think it is 2 minutes. The person asked to keep time should say: “it’s been two minutes.” The decision to do this can be made based on scheduling factors and the team’s capacity for performing compressions. If you think the cycles should be shortened, discreetly tell the person keeping time.

**Time = 2 min after stopping compressions (start when compressions are stopped NOT at ROSC)**

*RN provides the learner with post-arrest ECG if not already ordered* by the learner.

If a learner orders an ECG, provide it immediately after chest compressions are stopped.

**Time = ~3 min after stopping compressions**

*RN wraps up the case:*

“The cath lab (alt: MICU, CCU, pending the learner’s disposition plan) just called. They are ready for the patient. Is there anything else you want before we go?”

Ok to stop earlier if the learner appears done with the case:

“Anything else you want done at this time?”
