# Supplementary material for: Capstone Simulation: A Multipatient Simulation for Senior Emergency Medicine Residents
Source: MedEdPORTAL. 2023 Nov 9;19:11361. doi: 10.15766/mep_2374-8265.11361 (PMC10632183; doi:10.15766/mep_2374-8265.11361)
Supplement: Supplementary file 1 — Scenario 1.docxScenario 1 Setup and Prompts.docxScenario 1 Stimuli.pptxScenario 1 Skills Checklist.docxScenario 2.docxScenario 2 Setup and Prompts.docxScenario 2 Adult Stimuli.pptxScenario 2 Peds Stimuli.pptxScenario 2 Skills Checklist.docxScenario 3.docxScenario 3 Setup and Prompts.docxScenario 3 Skills Checklist.docxExample Schedule.xlsxDebriefing Material.docxPostsession Evaluation.docx [file mep_2374-8265.11361-s001.zip › E. Scenario 2.docx]

| **Appendix E: *Scenario 2***  **SIMULATION CASE TITLE: Capstone Case 2: ATLS for adult and pediatric patient**  **AUTHORS: Caitlin Schrepel, MD, Anne Chipman, MD, MS, Ross Kessler, MD, Crystal Phares, MD, Elizabeth Rosenman, MD**  **LEARNER AUDIENCE: PGY3 or PGY4 Emergency Medicine Residents** | |
| --- | --- |
| **PATIENT NAME:**  **Adult: Unknown**  **Pediatric patient: Timmy**  **PATIENT AGE:**  **Adult: Approximately 30 years old**  **Pediatric patient: 6 years old**  **CHIEF COMPLAINT: Two patients brought in after a motor vehicle collision. A 30-year-old male arrives intubated, while the 6-year-old arrives awake and stable with ankle pain.**  **PHYSICAL SETTING: Emergency department resuscitation bay with divider to create two rooms.** | |
|  | |
| **Brief narrative description of case** | *The learner arrives at the second simulated resuscitation bay and the embedded standardized nurse informs them that EMS has brought a young male, approximately 30 years old, who was involved in a highway speed motor vehicle collision and was intubated for mental status changes, but otherwise has normal vital signs. The learner walks into the resuscitation bay to see an intubated patient with one IV. The learner should proceed with ATLS. Primary survey shows right sided chest wall crepitus and diminished right sided breath sounds. The first chest radiograph will demonstrate a deep sulcus sign, concerning a pneumothorax. Approximately three minutes after arrival, the patient will become increasingly tachycardic, hypoxemic, and hypotensive. At this time, the second nurse will report the arrival of a second patient; a 6-year-old pediatric patient with ankle pain and a headache. After an initial primary survey of the pediatric patient (who is stable), the first nurse will inform the learner that the adult patient is hypotensive. At this point, the learner should initiate blood transfusion, call general surgery, and ask for a FAST exam. The FAST is positive for fluid in the right upper quadrant and lack of lung sliding on the right. After recognizing the pneumothorax, either by the initial chest XR or lack of lung sliding on US, the learner should indicate that a chest tube or needle decompression will need to be performed. The nurse will ask the learner to describe the procedure. Following this procedure, the patient’s oxygenation will improve, however he will remain hypotensive and tachycardic due to hemorrhagic shock and will need operative intervention.. During resuscitation of the adult patient, the pediatric patient will continue to complain of more and more ankle pain, until pain medications are ordered using weight-based dosing. Finally, after the adult patient is moved to the OR, the learner should complete a more thorough evaluation of the pediatric patient, obtain an ankle radiograph and discuss any additional management plans.* |
| **Primary Learning Objectives** | *By the end of this session, learners will be able to:*  *Demonstrate the ability to manage a hypotensive trauma patient using ATLS guidelines.*  *Identify and appropriately manage a pneumothorax in a hemodynamically unstable trauma patient.*  *Recognize and appropriately manage a positive FAST in a hemodynamically unstable trauma patient.*  *Apply ATLS guidelines to a stable pediatric patient.*  *Demonstrate the ability to lead a team while managing two simultaneous patients.*  *Prioritize tasks while managing two patients.* |
| **Critical Actions** | *Adult patient:*   1. *Assess ABCs on patient arrival.* 2. *Start blood transfusion for hypotensive patient.* 3. *Obtain and interpret a FAST exam in a hypotensive patient.* 4. *Recognize and manage pneumothorax.* 5. *Call general surgery and activate OR*   *Pediatric patient:*   1. *Assess ABCs on patient arrival.* 2. *Treat pain with weight-based pain medications* 3. *Complete secondary exam* 4. *Stabilize right ankle fracture* |
| **Learner Preparation or Prework** | *Learners were briefed on the following before Scenario 1:*  Environment: Community hospital  ---Community hospital with consultants available by phone.  ---OR, acute care, and ICU admissions available.  Team: Will be in the room, but you can ask for more resources as needed.  Simulation: Reminder of manikin capabilities. Reminder to ask the RN if there are any questions about fidelity or availability of resources.  Questions: Any resident questions were clarified. |

| **Initial Presentation (adult Patient)** | | | |
| --- | --- | --- | --- |
| **Initial vital signs** | **BP 105/77, HR 90, RR 12, Sat 95% intubated** | | |
| **Overall Setting and Appearance** | *When the learners enter the room they are in a simulated emergency department resuscitation bay. The patient is on the stretcher clothes with 1 peripheral IV placed. The airway cart, ventilator and IV polls are in the room just as they would be in a resuscitation room. The nurse is at the bedside on participant arrival. There is a divider in the room for a second patient’s room. The learner should not be able to see the second patient.* | | |
| **Standardized Participants (and their roles in the room at case start**) | *As the participant enters the room the paramedic will provide the initial history. The nurse states, “EMS brought us a young male, they’re guessing in his 30’s, involved in a highway speed MVC. There was extensive damage to the vehicle. He was agitated and then somnolent at the scene, so he was intubated for mental status changes. He was intubated with etomidate and rocuronium. He has been hemodynamically stable. No information on PMH/meds/allergies. He just arrived – RT transitioned him to the vent and we have him on the monitor, but no one has evaluated him yet and we don’t have any orders. It sounds like there may be another patient coming from the same accident.”* | | |
| **HPI** | Patient is intubated and not able to give additional history. | | |
| **Past Medical/Surgical History** | **Medications** | **Allergies** | **Family History** |
| *Unknown* | *Unknown* | *Unknown* | *Unknown* |
| **Physical Examination** | | | |
| **General** | Intubated, on a ventilator, wearing street clothes that have been cut by EMS | | |
| **HEENT** | Atraumatic, no facial trauma, pupils equal and reactive to light | | |
| **Neck** | No C-spine step offs | | |
| **Lungs** | Decreased breath sounds and chest wall crepitus on the right | | |
| **Cardiovascular** | Normal rate, no murmurs, palpable pulses | | |
| **Abdomen** | No distension, pt sedated without obvious tenderness | | |
| **Neurological** | Intubated and sedated (SP nurse can provide: “reported to be moving all extremities prior to intubation” if asked by learner) | | |
| **Skin** | No bruising, lacerations or rashes | | |
| **GU** | No bruising or hematoma, no blood at the meatus | | |

| **Initial Presentation (Pediatric Patient)** | | | |
| --- | --- | --- | --- |
| **Initial vital signs** | **HR 95 BP 95/70 O2 Sat 99% RR 20** | | |
| **Overall Setting and Appearance** | *When the learner enters the resuscitation bay, they should not be able to see this patient. Approximately 3 minutes after the arrival of the adult patient, the second nurse announces the arrival of the pediatric patient. When the learner enters the room, the pediatric patient is wearing a gown and lying in bed. He is scared but awake and alert.* | | |
| **Standardized Participants (and their roles in the room at case start**) | *The nurse in the second room tells the learner “we have a 6-year-old boy here from the same vehicle.” This nurse will prompt the learner to address the patient’s complaint of ankle pain.* | | |
| **HPI** | *You were in a booster seat in the back. You did not lose consciousness. Your only complaints are right ankle pain and head pain. You’re not sure if you hit your head. If asked about nausea you should ask: “What’s that?” You do feel like you might “get sick” but you haven’t vomited. The answer to most other symptoms (numbness, weakness, neck pain, belly pain, etc.) should be “no.”* | | |
| **Past Medical/Surgical History** | **Medications** | **Allergies** | **Family History** |
| *“I don’t know”* | *“I don’t think do”* | *“I don’t think so”* |  |
| **Physical Examination** | | | |
| **General** | Awake and alert, speaking normally, scared | | |
| **HEENT** | Atraumatic, no facial tenderness, pupils equal and reactive to light | | |
| **Neck** | No C spine tenderness, normal range of motion | | |
| **Lungs** | Equal, normal breath sounds, no respiratory distress | | |
| **Cardiovascular** | Normal rate and rhythm, no murmur, normal peripheral pulses | | |
| **Abdomen** | Soft and nontender, no bruising | | |
| **Neurological** | Normal | | |
| **Skin** | No lacerations or abrasions | | |
| **Muscular** | Right ankle tenderness and swelling with decreased ROM | | |

| **Instructor Notes - Changes and CASE Branch Points**  ***See Appendix F for additional instructions and prompts for this case.*** |
| --- |

**
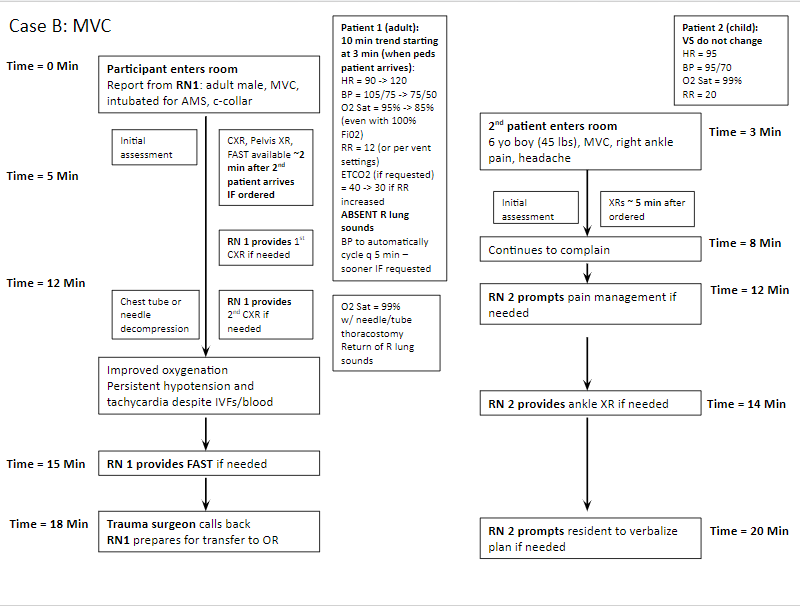
**

**Citation: Image is Author Created and Owned**

| Instructor Notes - Changes and Case Branch Points **FOR ADULT Patient** | | |
| --- | --- | --- |
| **Time Point** | **Change in Case/Actions** | **Additional Information** |
| *Time 0 min* | Adult pt: RN1 provides history with VS (BP 105/77, HR 90, RR 12, Sat 95% intubated)  If asked, RN provides this information: He has a single 18ga IV in place. He was seen moving all extremities prior to intubation. | *Adult pt history:* Young male, they’re guessing in his 30’s, involved in a highway speed MVC. There was extensive damage to the vehicle. He was agitated and then somnolent at the scene, so he was intubated for mental status changes – he was intubated with etomidate and rocuronium. He has been hemodynamically stable. No information on PMH/meds/allergies. He just arrived  RT transitioned him to the vent, and we have him on the monitor, but no one has evaluated him yet and we don’t have any orders. |
| *Time 0 - 3 min* | Participant to start a primary survey of the adult patient.  CXR, Pelvis XR, and FAST exam are available 2 min after ordered. | Sim manikin should be set to have absent lung sounds on the right. |
| *Time 3 min* | Start 10 min vital sign trend for adult patient: Tech begins vital sign trend as noted on the right side of the flow chart above (worsening tachycardia, hypotension, hypoxia). | This should occur at the same time that the pediatric patient arrives in the room next door, but not before.  Generally the participant will evaluate the pediatric patient immediately on their arrival and then return to the adult patient after RN1 becomes concerned about the vital signs. |
| *Time ~5-8 min* | RN1 should prompt the participant to return to the adult patient with information that they are hypotensive and hypoxemic. | Ideally this will occur after the participant has completed the primary survey for the pediatric patient. However, this timeframe should be followed regardless of where the participant is with the pediatric patient. |
| *Time 3 min -13 min* | Participants should search for the cause of hypotension and hypoxemia and begin treatment.  This patient has 2 problems:   1. Right sided pneumothorax→ After resident verbalizes needle decompression/chest tube placement, hypoxemia should resolve. 2. Hemoperitoneum→ IVF and/or blood will not resolve hypotension. The pt should remain hypotensive regardless of participant action to prompt emergent OR. | If no 1st CXR was ordered by 5 min, the RN should order it.  If no 2nd CXR was ordered by 2 min after chest tube placement/decompression, RN should order it.  At ~12 min RN1 should prompt recognition of the pneumothorax:  (e.g., “I’m not hearing breath sounds on the right.”)  Blood products willimprove the vital signs, but donot fully correct the blood pressure to normal. |
| *Time 15 min* | RN1 provides FAST if never ordered:  “I asked radiology to do a FAST while you were with the kid. The images are up on the computer now.” |  |
| *Time 18 min* | Trauma surgeon calls in for a report to help wrap up the case. | Potential discussions with the participant:   1. If a chest tube is in place and blood products have been started: Accept the patient to the OR. 2. If a chest tube is in place, but blood products have not been started: Ask the participant about the results of the FAST. If they recognize the hemoperitoneum, then ask if they have blood available they can start. If they do not recognize the hemoperitoneum, you can interpret the FAST for them and ask them to start blood. 3. If blood products have been started, but pneumothorax treated only with needle decompression: Ask the participant to place a chest tube for transport. 4. If blood products have been started, but the pneumothorax has not been treated: Ask the participant why the patient is still hypoxemic. If they do not recognize the pneumothorax, you should say radiology called you with the result (no obvious pneumothorax, but has deep sulcus sign and subcutaneous air). 5. If neither concern has been addressed: You should interpret the FAST for the participant and ask them to place a chest tube and start blood. |
| *Time 20 min* | RN1 wraps up the case and prompts the learner to verbalize a plan for pediatric patient if needed.  “The OR is calling for him. Anything else for the kiddo right now?” |  |

| Instructor Notes - Changes and Case Branch Points **FOR PEDIATRIC Patient** | | |
| --- | --- | --- |
| **Time Point** | **Change in Case/Actions** | **Additional Information** |
| *Time 0* | Pediatric patient not yet arrived |  |
| *Time 3 min* | Pediatric patient arrives in the second room.  Vital signs of pediatric patient do not change during the case (HR 95 BP 95/70 O2 Sat 99% RR 20) | RN2 announces: “We have a 6-year-old boy here from the same vehicle.”  If asked, you should inform the learner that no more patients are expected from this collision.  Pediatric patient (see additional instructions on pediatric role in Appendix F): only complaint is right ankle pain and, if specifically asked, a little head pain. No medical problems or medications. Doesn’t know about allergies.  Should ask about his father:  “Is my dad here? Is he going to be ok?” |
| *Time 3 min-8 min* | Participant starts primary and secondary survey of the pediatric patient.  CXR, Pelvic XR, and ankle XR available ~5 min after ordered. | During evaluation of the pediatric patient, the RN for the adult patient will announce worsening vital signs.  This is generally done after the primary survey of the pediatric patient, but will depend on how quickly the participant works.  It is expected that the participant will return to the pediatric patient to finish their evaluation at some point. The prompts below can be used as needed. |
| *Time 8 min* | Pediatric patient becomes more vocal about RLE pain.  “Ouch, my ankle hurts!”  “Where is my dad?” |  |
| *Time 12 min* | RN2 prompts pain management if needed. |  |
| *Time 14 min* | RN2 provides ankle XR on the child if it wasn’t ordered: “His ankle is really hurting him, and you were busy, so I went ahead and ordered a XR. I hope that’s OK.” |  |
| *Time 20 min* | RN1 wraps up the case and prompts the learner to verbalize a plan for pediatric patient if needed.  “The OR is calling for him. Anything else for the kiddo right now?” |  |

**Ideal Scenario Flow**

*Adult patient:* The scenario will start with RN1 informing the participant that EMS brought in an approximately 30 year-old-male who was involved in a motor vehicle collision and intubated in the field. On arrival the patient will have normal vital signs and the participant will begin the primary survey. On primary survey they will note diminished right sided breath sounds and normal vital signs. They will confirm IV access and ask for a second IV, in addition to ordering a CXR and a pelvis XR. About 2 minutes after the verbal order, the CXR and pelvis XR will be available. The participant will recognize a deep sulcus sign, concerning for a pneumothorax. They will verbalize the need for needle decompression or chest tube. Approximately three minutes after arrival, the patient will become increasingly tachycardic, hypoxemic, and hypotensive. At this point, if not done already, they will perform needle decompression and/or chest tube placement. RN1 will ask them to describe the procedure. Once appropriately described, hypoxemia will resolve but the patient will remain hypotensive and tachycardic. The participant will order STAT blood products in a 1:1 ratio, call general surgery, ask for a FAST, and repeat the CXR. Repeat CXR will show placement of the chest tube and FAST will demonstrate fluid in the right upper quadrant. Once hemorrhagic shock and the need for the OR is recognized, the trauma surgeon will call in to the room and accept the patient to the OR.

*Pediatric patient:* Three minutes after arrival of the adult patient, RN2 will report the arrival of a second patient; a 6-year-old pediatric patient with ankle pain and a headache. This patient will remain stable throughout treatment. The participant will quickly perform a primary survey on this patient, and ask for any initial orders to be placed before being called to take care of the worsening adult patient. Ideally, the participant will order a CXR, pelvis XR, and ankle XR in this patient although acceptable imaging options could vary. They will also order weight-based pain medications and provide reassurance to the child. If pain medication was not initially provided, the pediatric patient will continue to complain of more pain. After the adult patient is moved to the OR, the participant will complete a more thorough evaluation of the pediatric patient and discuss next steps in management.

**Anticipated Management Mistakes**

1. Trouble recognizing the pneumothorax on CXR: We have found that several participants had trouble recognizing the pneumothorax on the provided CXR. The CXR demonstrates a deep sulcus sign and abundant subcutaneous air, but a pleural line is not visible . For this reason, some participants were hesitant to place the chest tube. If this occurs, it can be helpful to do one of two things. RN1 can ask what the CXR demonstrated. This tends to get the participants to look a little closer at the image at which point they recognize these findings. If this does not prompt recognition of the pneumothorax, RN1 can ask what the ultrasound of the lungs looked like, which prompts them to key in on looking for lung sliding. Importantly, if the participant places a chest tube before obtaining the FAST, the side with absent lung sliding should not be shown. Instead the side with normal lung sliding can be shown twice.

2. Early completion of interventions: Some participants quickly identify the pneumothorax and hemoperitoneum of the adult patient, and treat them accordingly. Because the vital signs of the adult patient do not normalize completely with interventions, the participant believes that they have missed something. It is okay to allow them to think about alternative causes such as neurogenic shock or bleeding in the pelvis. However, if they tinker too long they may make management mistakes. Once they have completed the appropriate treatment, it is okay for the trauma surgeon to call in early to help wrap up the case and accept the patient to the OR.

3. Mis-dosing medications for the pediatric patient: Several participants tried to guess on dosing for pain medications for the pediatric patient. If the dose they provide is not correct, we recommend having RN2 state that they “confirmed a dose with the pharmacy and changed the order.” The participant won’t get credit for this on the check-list, but this avoids the simulation going off course because of an accidental overdose of pain medications.
