# Supplementary material for: Capstone Simulation: A Multipatient Simulation for Senior Emergency Medicine Residents
Source: MedEdPORTAL. 2023 Nov 9;19:11361. doi: 10.15766/mep_2374-8265.11361 (PMC10632183; doi:10.15766/mep_2374-8265.11361)
Supplement: Supplementary file 1 — Scenario 1.docxScenario 1 Setup and Prompts.docxScenario 1 Stimuli.pptxScenario 1 Skills Checklist.docxScenario 2.docxScenario 2 Setup and Prompts.docxScenario 2 Adult Stimuli.pptxScenario 2 Peds Stimuli.pptxScenario 2 Skills Checklist.docxScenario 3.docxScenario 3 Setup and Prompts.docxScenario 3 Skills Checklist.docxExample Schedule.xlsxDebriefing Material.docxPostsession Evaluation.docx [file mep_2374-8265.11361-s001.zip › F. Scenario 2 Setup and Prompts.docx]

**Appendix F: Scenario 2 Set-up and Prompts**


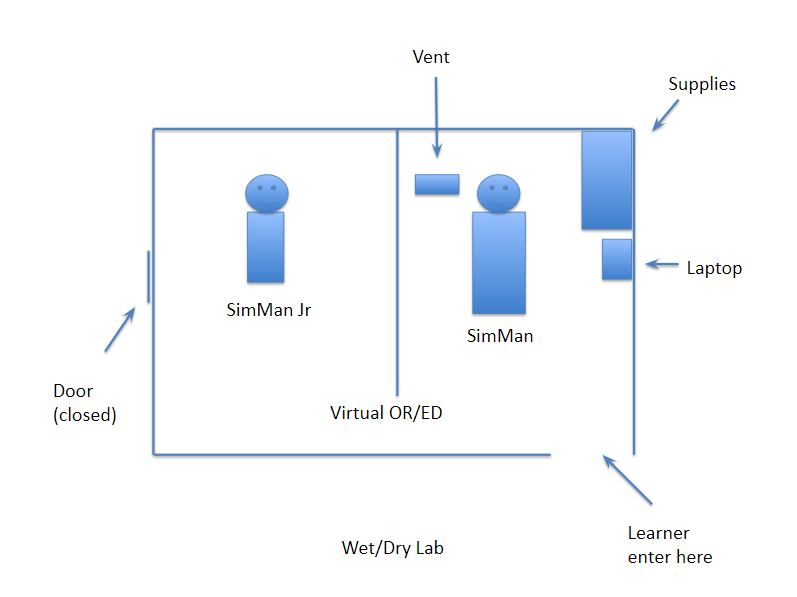


**Citation: Image is Author Created and Owned**

Adult Mannequin Set-up:– High-technology human patient simulator (no voice needed).

- - Covered in shirt and pants (already cut to facilitate removal)
- Right chest wall crepitus: tegaderm packets of rice crisp cereal placed underneath the chest wall skin of the manikin.
- Intubated with 7.5 ETT @ 22 cm.
  - Single 18 ga IV in place
  - Monitor
- Monitor:
  - Telemetry, BP cuff, pulse oximetry hooked up.
  - Monitor on
  - Initial VS: HR 90 in sinus rhythm, BP 105/77, RR 12, Sat 95%
  - Absent breath sounds on right.
    - No alarm sounds.

Pediatric Mannequin Set-up – High-technology human patient simulator (needs voice--e.g., faculty instructor, sim center staff, volunteer)

- - No backboard or c-collar.
  - In patient gown
  - No IV – won’t push fluids but will have IV “patch” setup (Coban and peripheral IVs) off to the side if learner requests an IV
  - Monitor
    - Initial VS: HR 100 in sinus rhythm, BP 90/50, RR 18, Sat 99% - static throughout
    - Not on monitor – must be requested by the participant.
    - No alarm sounds.

Additional Equipment:

- Room divider between adult and pediatric simulation manikin– see diagram.
- Code cart – in room
  - Self-inflating bag and mask
  - Mac 3 or 4 laryngoscope blade and handle
  - 1 opened ETCO2 detector
  - OG/NG tube
  - Two packs of RBCs
  - Two packs of FFP
  - Tape
  - Penlight
  - Broselow tape
- Ventilator (does not need to be working)
- Box splints – small
- Towels (4)
- Laptop for images
- Telephone for calling/receiving calls from the control room.

AV Needs:

- Microphone for learner
- Microphone for faculty member to provide pediatric patient voice.
- Radio with earpiece for SP nurse
- Radio in control room to communicate with SP nurse.

*Prompt times are approximate and should be used as guidelines to keep the case and participant on track.*

Primary roles: RN1, RN2, Pediatric Voice

Additional roles: 1-2 – MAs

Sim Technician

Faculty Instructor

Both RNs start in the room. RN1 gives report.

2 additional personnel are available upon request (e.g., MA and/or RT)

**Time = 0 min**

*RN1 provides history* and triage sheet with VS (BP 105/77, HR 90, RR 12, Sat 95% intubated)

**History:**

Young male, they’re guessing in his 30’s, involved in a highway speed MVC. There was extensive damage to the vehicle. He was agitated and then somnolent at the scene, so he was intubated for mental status changes – he was intubated with etomidate and rocuronium. He has been hemodynamically stable. No information on PMH/meds/allergies. He just arrived – RT transitioned him to the vent, and we have him on the monitor, but no one has evaluated him yet and we don’t have any orders. It sounds like there may be another patient coming from the same accident.

*Additional history – provide only if requested:*

*You can answer questions about mechanism details (restrained, airbag deployment, etc.).Keep details consistent between cases.*

He has a single 18ga IV in place. He was seen moving all extremities prior to intubation.

**Time = 3 min**

*RN2 reports arrival of second patient:*

“We have a 6-year-old boy here from the same vehicle.”

If asked, you can inform the learner that no more patients are expected from this collision.

*Pediatric patient* (see additional instructions on pediatric role play below): only complaint is right ankle pain and, if specifically asked, a little head pain. No medical problems or medications. Doesn’t know about allergies. Should ask about his father:

“Is my dad here? Is he going to be ok?”

Tech begins VS trend for the adult patient (worsening tachycardia, hypotension, hypoxia).

**Time > 5 min**

Imaging for adult patient will be available ~2 min after ordered (CXR, pelvis XR, FAST)

**Time = 8 min**

*Pediatric patients become more vocal* about RLE pain.

*RN1 provides 1st CXR on adult patient if it wasn’t already ordered* (no obvious pneumothorax, but has deep sulcus sign and subcutaneous air)

**Time = 12 min**

*Pediatric patient* complains of pain.

*RN2 prompts* pain management for pediatric patient if needed.

*RN1 prompts identification of pneumothorax if needed* (e.g., “I’m not hearing breath sounds on the right.”)

**Time = 14 min**

*RN2 provides ankle XR on child if it wasn’t ordered.*

“His ankle is really hurting him, and you were busy, so I went ahead and ordered a XR. I hope that’s OK.”

**Time = 15 min**

*RN1 provides FAST if never ordered.*

“I asked radiology to do a FAST while you were with the kid. The images are up on the computer now.”

**Time = 18 min**

*Trauma surgeon (e.g., faculty instructor) calls in.*

**Time = 20 min**

*RN1 wraps up the case* and prompts the learner to verbalize a plan for pediatric patient if needed.

“The OR is calling for him. Anything else for the kiddo right now?”

After the learner answers, the team should then get the adult patient ready for transfer (e.g., switch to portable oxygen, portable monitor, bagging, etc.). It is important to look busy even if you are not, otherwise the learner will think they have missed something specific and will continue to call out orders and will be hesitant to move on to the next case.

**Diagnostics:**

Adult:

Blood glucose – available immediately (normal)

ECG, CXR, pelvis XR, FAST all available ~2 min after ordered AND 2nd patient arrives (whichever occurs later)

(CXR provided at 12 min and FAST provided at 15 min if not ordered)

Pediatric:

Ankle XR, CXR, pelvis XR all available ~5 min after ordered.

(ankle XR provided at 14 min if not ordered)

**Pediatric Trauma Patient Voice Guidelines**

HPI:

You were in a booster seat in the back. You did not lose consciousness. Your only complaints are right ankle pain and a head pain. You’re not sure if you hit your hit. If asked about nausea you should ask: “What’s that?” You do feel like you might “get sick” but you haven’t vomited. The answer to most other symptoms (numbness, weakness, neck pain, belly pain, etc.) should be “no.”

PMH/MEDS:

“I don’t think so.”

Allergies:

“I don’t know.”

SH:

You live with your parents. If they ask where your mom is, respond: “Probably working.” If they ask where she works, respond: “At a school. She’s a teacher” (but you don’t know which school).

Physical Exam:

Pay attention to the physical exam. Since the manikin can’t move, you will need to give verbal clues to indicate a normal exam (example: “yup, my arms are just fine”). You have a mild headache if specifically asked, but no pain on palpation (“It just hurts.”) The only abnormality on the physical exam is pain in your right ankle. This will hurt too much to move, but you can verbalize a normal sensation (“yes, I can feel that”) and “wiggle your toes” verbally.

Prompts:

Use your judgment – we want them to prioritize and task switch, but we don’t want to go overboard trying to distract them.

As the case progresses you should become more vocal about your pain to prompt pain management and imaging:

Complain of pain on arrival, at 8 min, and at 12 min

You can stop after the learner does ALL of the following:

- Performs a physical exam.
- Obtains vital signs.
- Orders pain meds
- Addresses your ankle (e.g., confirms pulses, orders x-ray, places some type of splint)

If the learner does all of the above early on, you can ask questions about how your dad is doing.
