# Supplementary material for: Capstone Simulation: A Multipatient Simulation for Senior Emergency Medicine Residents
Source: MedEdPORTAL. 2023 Nov 9;19:11361. doi: 10.15766/mep_2374-8265.11361 (PMC10632183; doi:10.15766/mep_2374-8265.11361)
Supplement: Supplementary file 1 — Scenario 1.docxScenario 1 Setup and Prompts.docxScenario 1 Stimuli.pptxScenario 1 Skills Checklist.docxScenario 2.docxScenario 2 Setup and Prompts.docxScenario 2 Adult Stimuli.pptxScenario 2 Peds Stimuli.pptxScenario 2 Skills Checklist.docxScenario 3.docxScenario 3 Setup and Prompts.docxScenario 3 Skills Checklist.docxExample Schedule.xlsxDebriefing Material.docxPostsession Evaluation.docx [file mep_2374-8265.11361-s001.zip › J. Scenario 3.docx]

| **Appendix J: Scenario 3**  **SIMULATION CASE TITLE: Capstone Case 3: Breaking bad news (simulated patient)**  **AUTHORS: Caitlin Schrepel, MD, Anne Chipman, MD, MS, Ross Kessler, MD, Crystal Phares, MD, Elizabeth Rosenman, MD**  **LEARNER AUDIENCE: PGY3 or PGY4 Emergency Medicine Residents** | |
| --- | --- |
| **PATIENT NAME: Dave**  **CHIEF COMPLAINT: Thomas’s (patient from Scenario 1) father presenting after being told his son is in the emergency department.**  **PHYSICAL SETTING: Simulated family room/social work room** | |
|  | |
| **Brief Narrative Description of Case** | *A family member has arrived and is waiting to hear an update about his son, who was the patient from Scenario 1 that sustained a STEMI and VF arrest. The patient subsequently died in the cath lab. The learner is informed of this outcome after finishing Scenario 2 and is asked to disclose this information to the patient’s father. The learner will be brought to a room where a standardized patient will play the father of patient 1. The learner will need to establish the relationship between the two individuals, ascertain what they already know, deliver the bad news, and leave the room after providing support.* |
| **Primary Learning Objectives** | *By the end of this session, learners will be able to:*  *Illustrate communication techniques to deliver bad news while avoiding euphemisms or confusion.*  *Demonstrate compassionate communication while delivering bad news.*  *Provide emotional support to a family member of a patient who has died in the emergency department.* |
| **Critical Actions** | 1. *Establish the relationship between the patient and the family member* 2. *Deliver the news that the patient’s son died in the cath lab* 3. *Uses appropriate compassionate communication and body language* 4. *Avoids medical jargon and euphemisms* 5. *Answers family member’s questions* 6. *Provides resources for next steps* |
| **Learner Preparation or Prework** | *Learners were briefed on the following before Scenario 1:*  Environment: Community hospital  ---Community hospital with consultants available by phone.  ---OR, acute care, and ICU admissions available.  Team: Will be in the room, but you can ask for more resources as needed.  Simulation: Reminder of manikin capabilities. Reminder to ask the RN if there are any questions about fidelity or availability of resources.  Questions: Any resident questions were clarified. |

| **Initial Presentation** | |
| --- | --- |
| **Overall Setting and Appearance** | *The standardized patient is waiting in the simulated family or social work room. There should be two chairs facing each other in the room.* |
| **Standardized Participant** | Role of the Standardized Participant:  You are the father of Thomas. Your name is Dave (alternatively, you can use your real name if you are comfortable with that so as to avoid confusion). You were called at home and told your son collapsed at work and was taken to the Emergency Department. You were NOT informed that he underwent CPR, that he had a heart attack, or that he died. Your wife (the patient’s mother) is currently out of town visiting family. The patient is not married and has no children.  Your role is to convey shock and grief. You should not become angry or overly dramatic. As noted above, you were unaware of the severity of your son’s condition when you arrived and therefore are not particularly concerned or upset when the learner first enters the room. A lot of what you say will depend on what the learner says to you so it is important you remain flexible. |
