# Supplementary material for: Capstone Simulation: A Multipatient Simulation for Senior Emergency Medicine Residents
Source: MedEdPORTAL. 2023 Nov 9;19:11361. doi: 10.15766/mep_2374-8265.11361 (PMC10632183; doi:10.15766/mep_2374-8265.11361)
Supplement: Supplementary file 1 — Scenario 1.docxScenario 1 Setup and Prompts.docxScenario 1 Stimuli.pptxScenario 1 Skills Checklist.docxScenario 2.docxScenario 2 Setup and Prompts.docxScenario 2 Adult Stimuli.pptxScenario 2 Peds Stimuli.pptxScenario 2 Skills Checklist.docxScenario 3.docxScenario 3 Setup and Prompts.docxScenario 3 Skills Checklist.docxExample Schedule.xlsxDebriefing Material.docxPostsession Evaluation.docx [file mep_2374-8265.11361-s001.zip › K. Scenario 3 Setup and Prompts.docx]

**Appendix K: Scenario 3 Step-up and Prompts**

Room:

- Small office

Set-up:

- Three chairs - 2 facing each other (participant and standardized patient) and one off to the side (observer)
- Box of tissues
- Hand sanitizer

**Background Information:**

Thomas is a 43-year-old man who collapsed while walking to lunch. His co-workers performed CPR and he had a pulse when EMS arrived. Upon arrival at the Emergency Department he became very hypoxemic (low oxygen level), requiring intubation. He then lost pulses again and CPR was started. The medical team was able to revive him, and testing revealed that he had a heart attack, and he was transferred to the “cath lab” for evaluation of his heart where he again went into cardiac arrest and died.

**Role of the Standardized Participant:**

You are the father of Thomas. You can use the name Dave, or you can use your real name if you are comfortable.. You were called at home and told your son collapsed at work and was taken to the Emergency Department. You were NOT informed that he underwent CPR, that he had a heart attack, or that he died. Your wife (the patient’s mother) is currently out of town visiting family. The patient is not married and has no children.

Your role is to convey shock and grief. You should not become angry or overly dramatic. As noted above, you were unaware of the severity of your son’s condition when you arrived and therefore are not particularly concerned or upset when the learner first enters the room. A lot of what you say will depend on what the learner says to you, so it is important you remain flexible. As best you can, please keep your responses consistent between participants. Appropriate reactions and questions include:

- Pausing upon hearing the news - prolonged pause if necessary, until the learner restarts the conversation.
- Asking for clarification – especially if the learner uses overly detailed (e.g., medical terminology) or vague language (e.g., euphemisms such as “he passed”)
- Repetitive statements or questions (“I don’t understand how this could happen” or “How could this happen?”)
- Becoming visibly upset (tremulous, tearful, voice changes – as per your comfort level)

In general, avoid prompting the learner. However, you may offer the following prompts if needed:

- If the learner only communicates what happened in the Emergency Department and tells you the patient was transferred to the “cath lab” but does not tell you he died you should specifically ask how he is doing (Example: “How is he doing now?” or “Is he going to be okay?”).
- If the learner is struggling to wrap things up you can ask: “What happens next?”

You will be asked to complete a brief assessment of the learner’s performance at the end of each session. Please look this over ahead of time so you know what you will be assessing the learner on.

**Additional Information:**

A “nurse” will be present during the scenario. This person is actually an observer/evaluator and, aside from starting and ending the scenario, will be minimally involved. The scenario will be recorded and reviewed by Emergency Medicine physicians for the purpose of evaluating and providing feedback to the learners.
