# Supplementary figures and images for: Capstone Simulation: A Multipatient Simulation for Senior Emergency Medicine Residents
Source: MedEdPORTAL. 2023 Nov 9;19:11361. doi: 10.15766/mep_2374-8265.11361 (PMC10632183; doi:10.15766/mep_2374-8265.11361)

## Slide 1
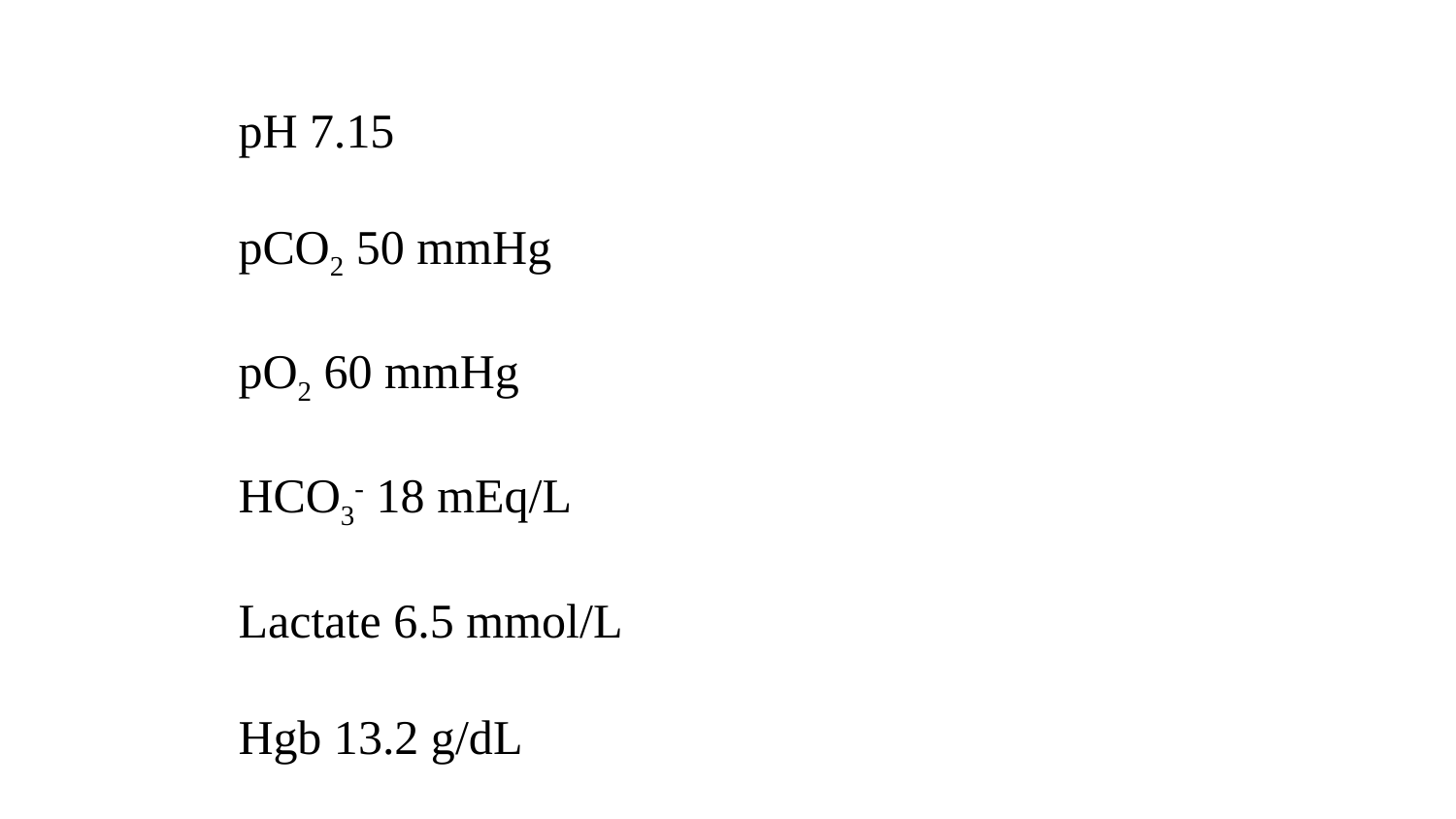

pH 7.15
pCO2 50 mmHg
pO2 60 mmHg
HCO3- 18 mEq/L
Lactate 6.5 mmol/L
Hgb 13.2 g/dL

## Slide 2
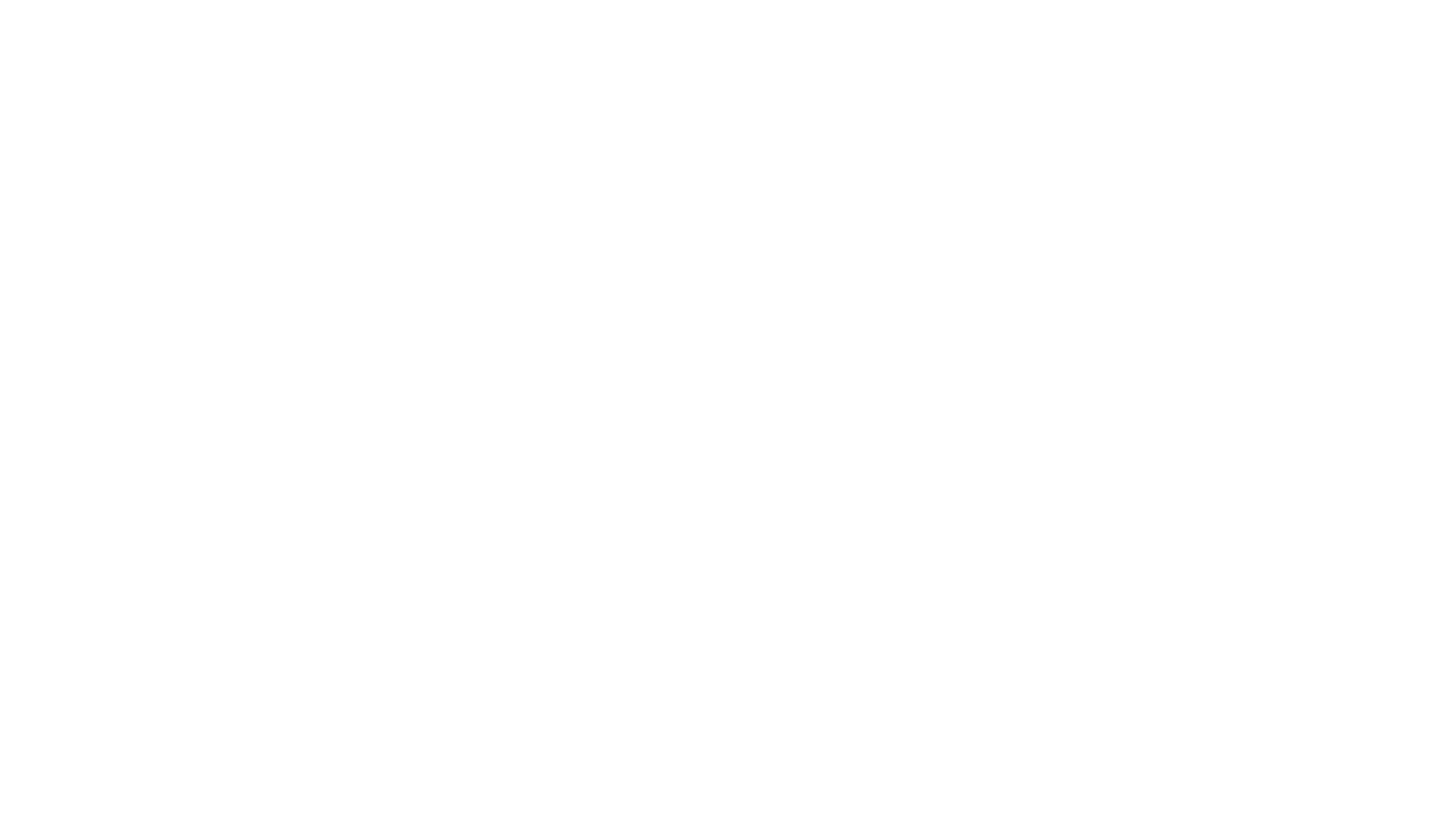

## Slide 3
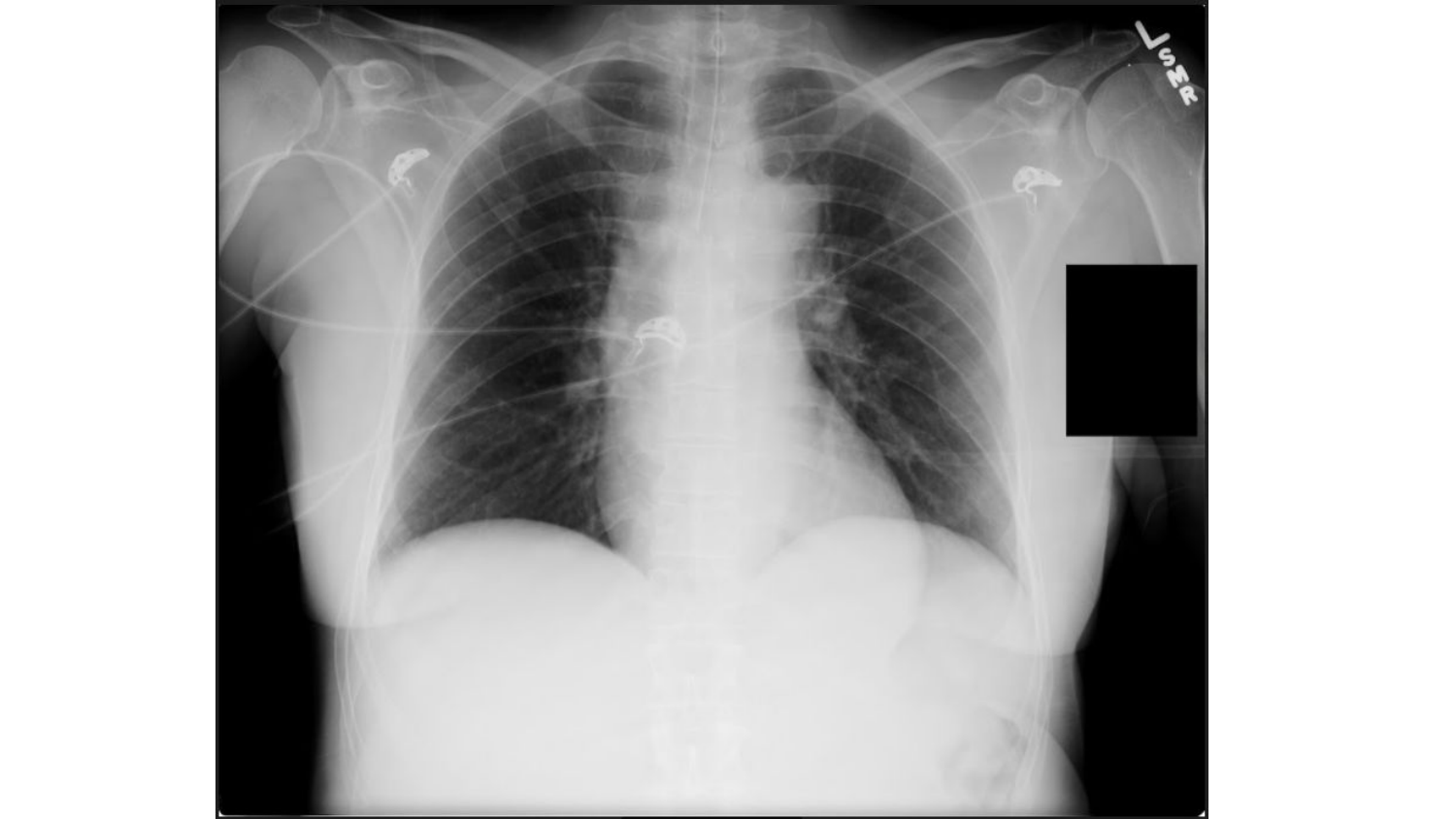

## Slide 4
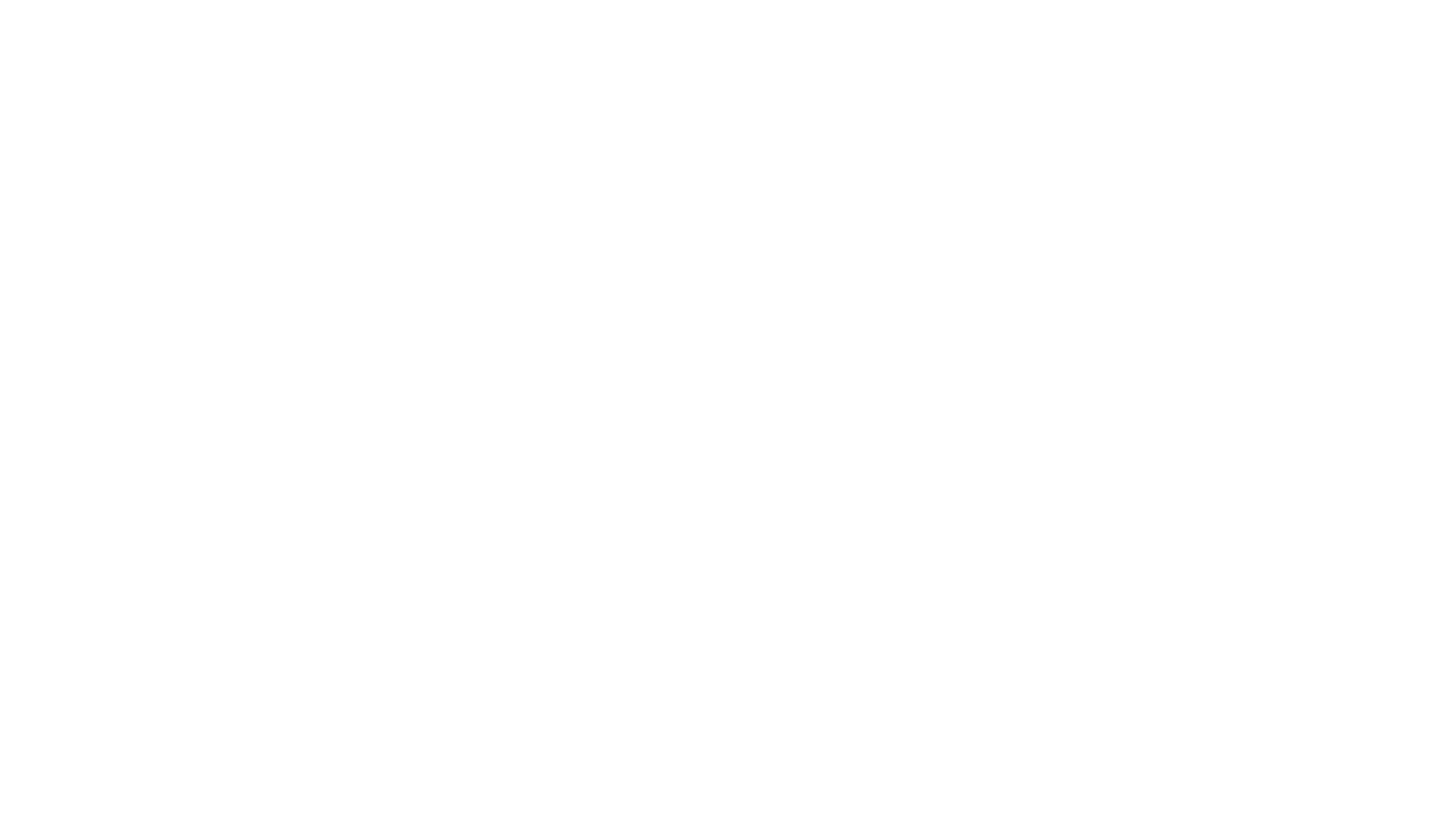

#

## Slide 5
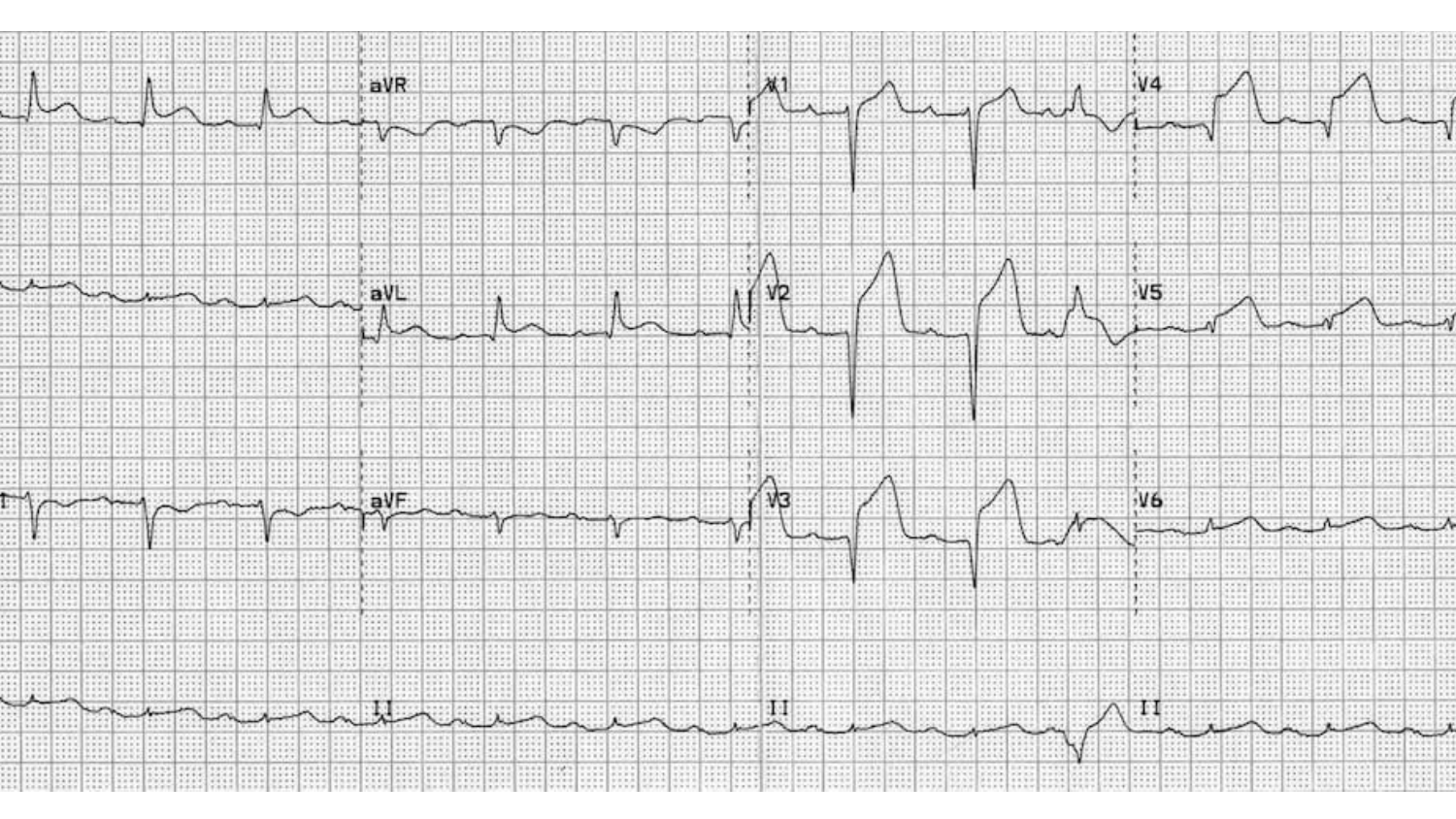

Supplement: Supplementary file 1 — Scenario 1.docxScenario 1 Setup and Prompts.docxScenario 1 Stimuli.pptxScenario 1 Skills Checklist.docxScenario 2.docxScenario 2 Setup and Prompts.docxScenario 2 Adult Stimuli.pptxScenario 2 Peds Stimuli.pptxScenario 2 Skills Checklist.docxScenario 3.docxScenario 3 Setup and Prompts.docxScenario 3 Skills Checklist.docxExample Schedule.xlsxDebriefing Material.docxPostsession Evaluation.docx [file mep_2374-8265.11361-s001.zip › C. Scenario 1 Stimuli.pptx]

## Slide 1
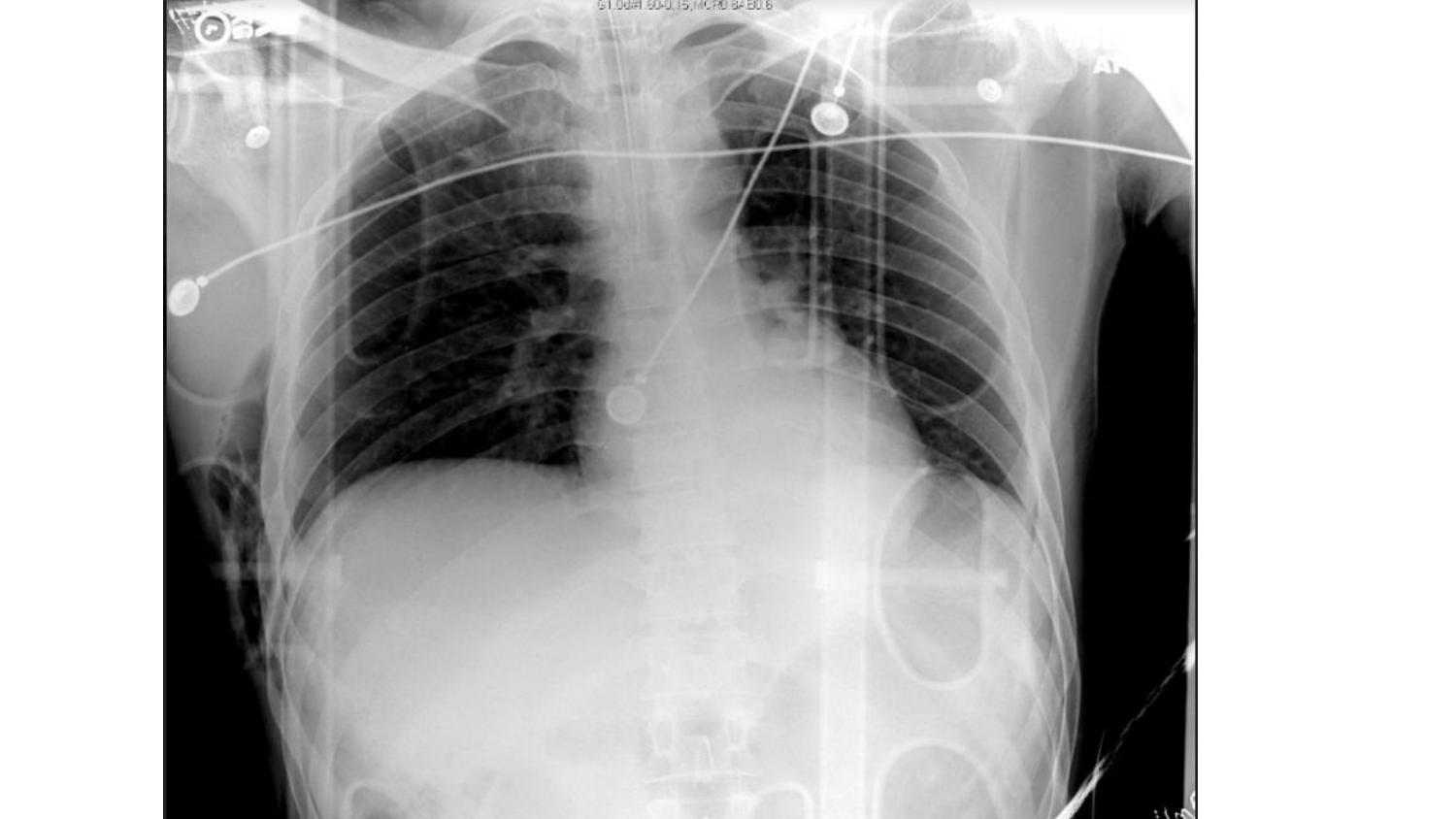

## Slide 2
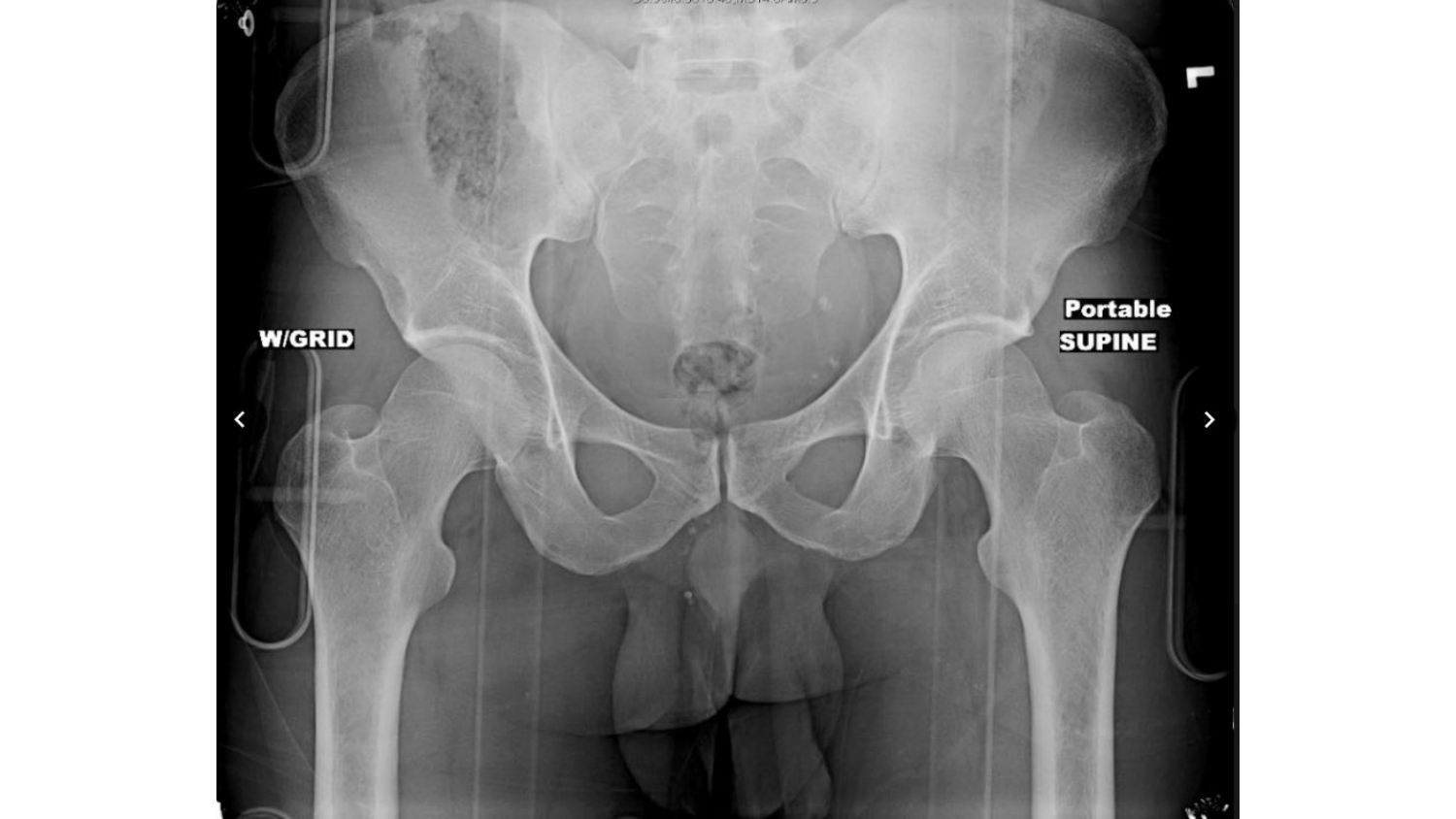

## Slide 3
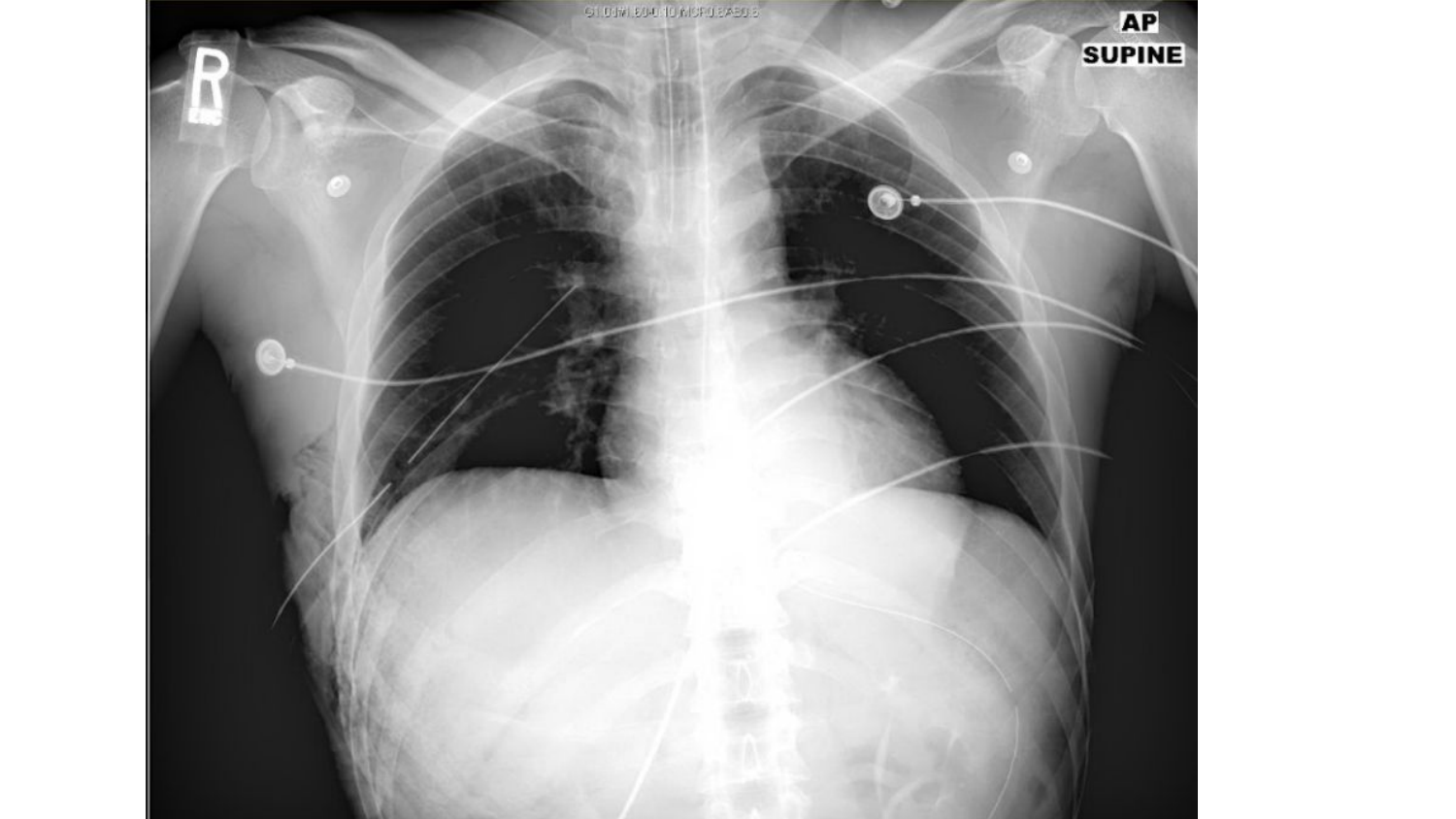

## Slide 4
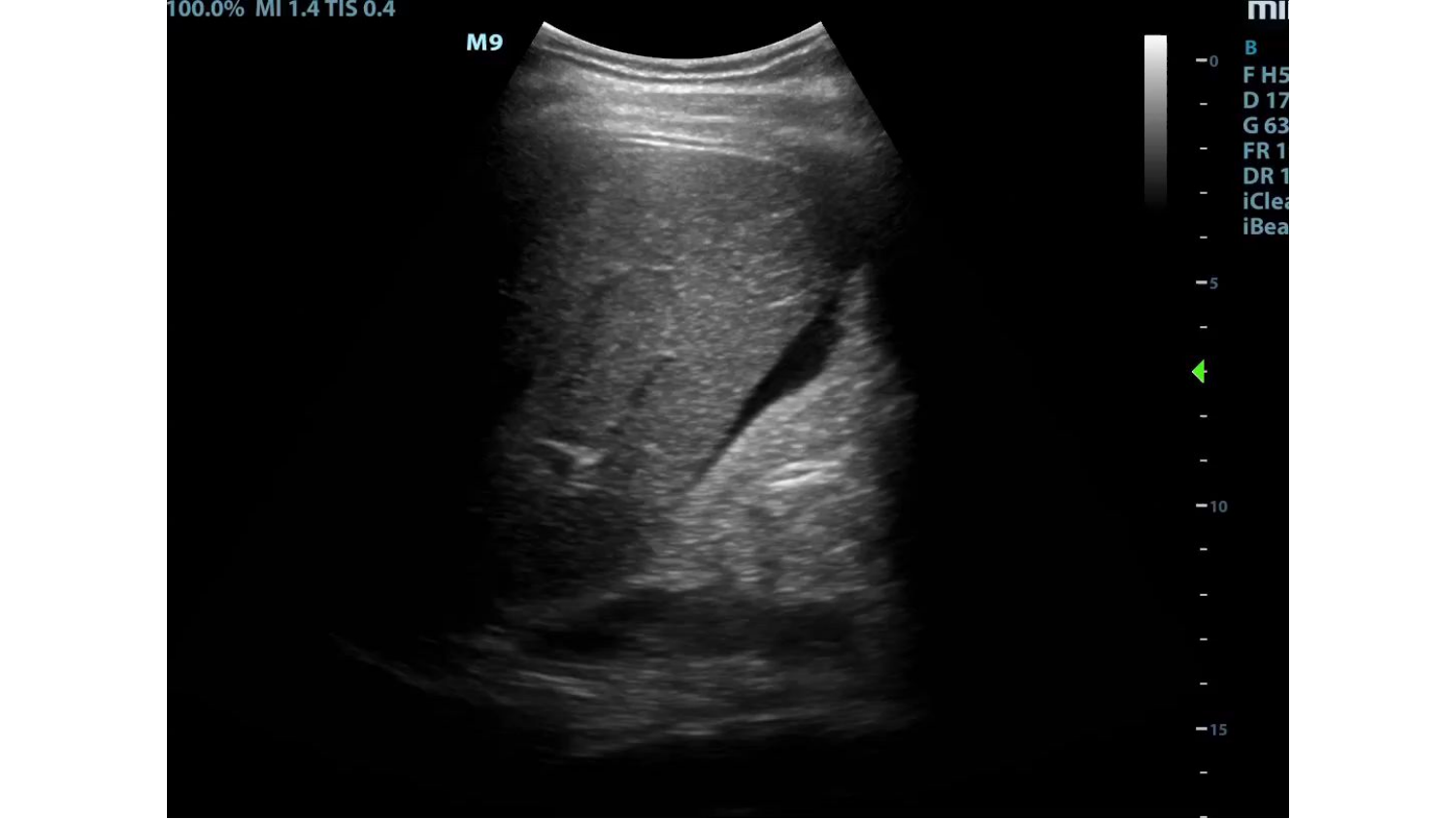

## Slide 5
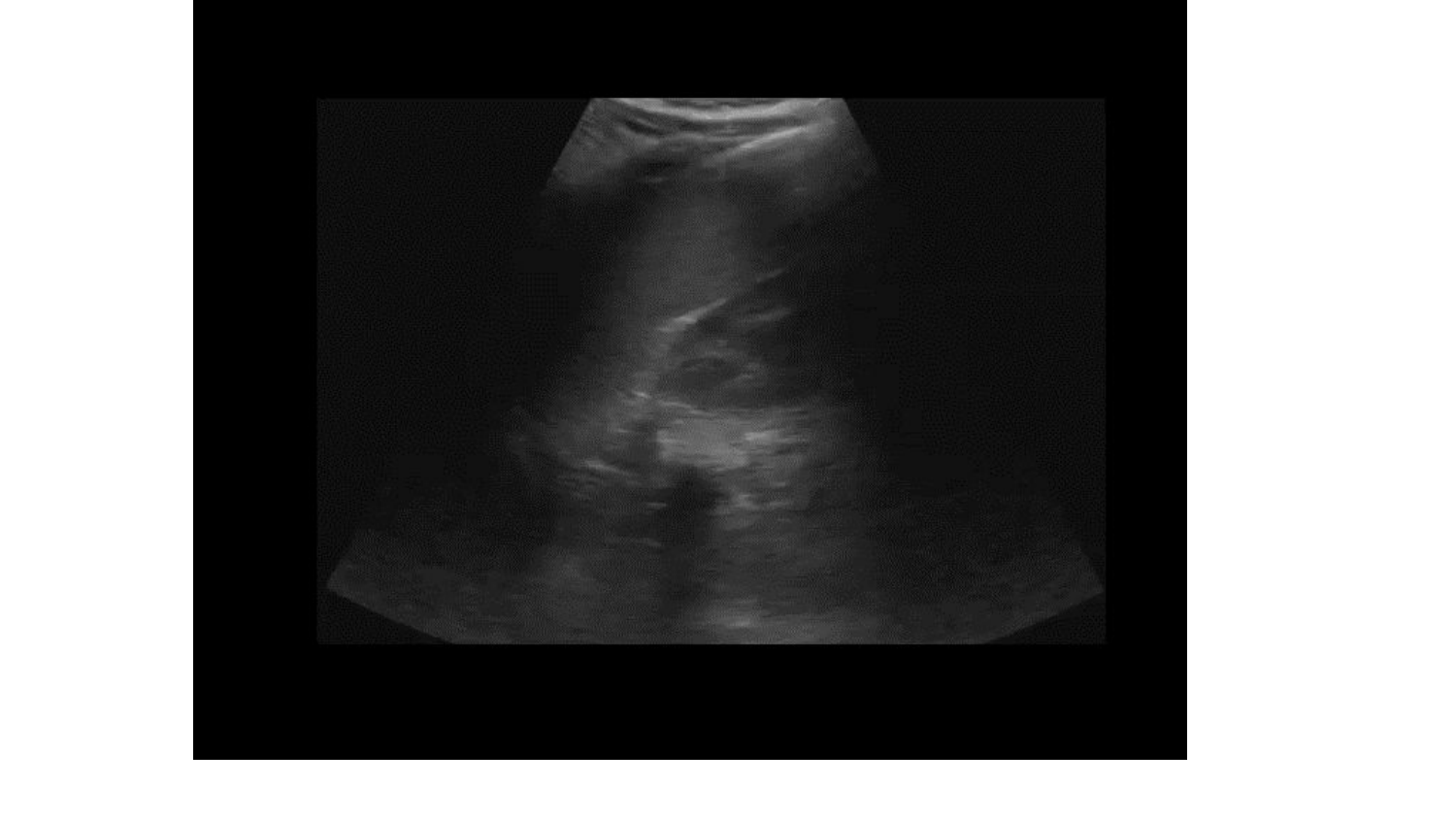

## Slide 6
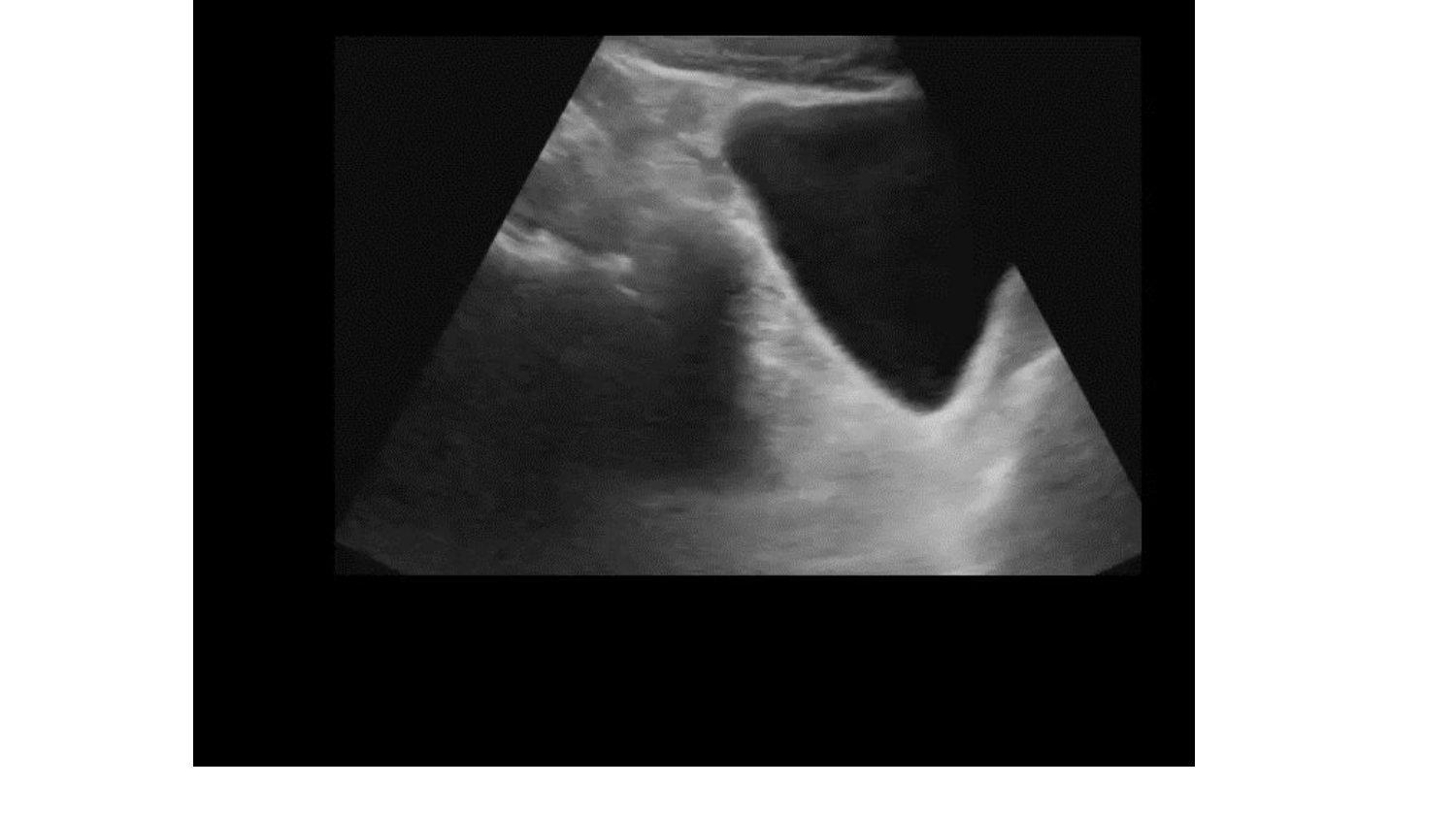

## Slide 7
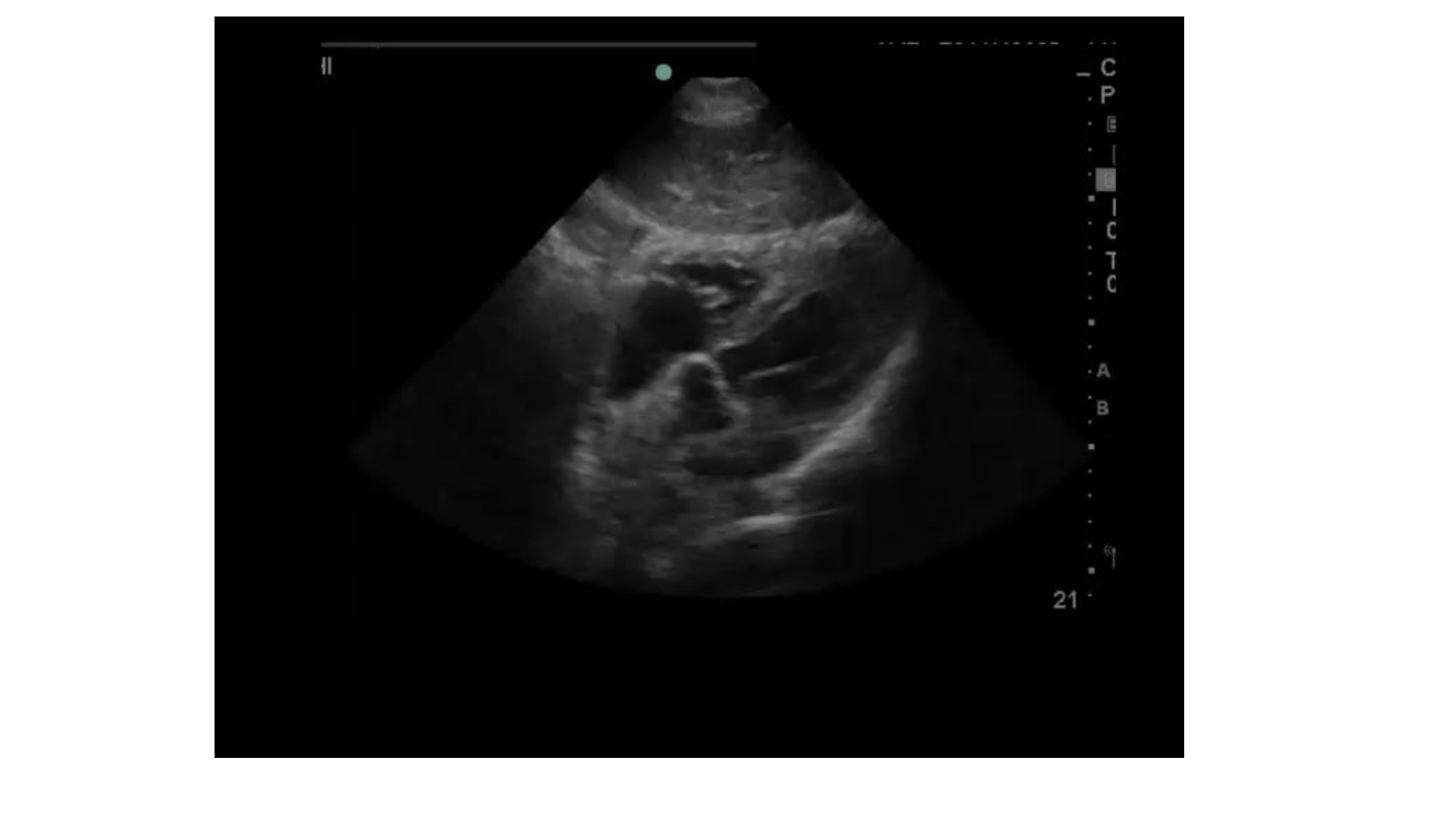

## Slide 8
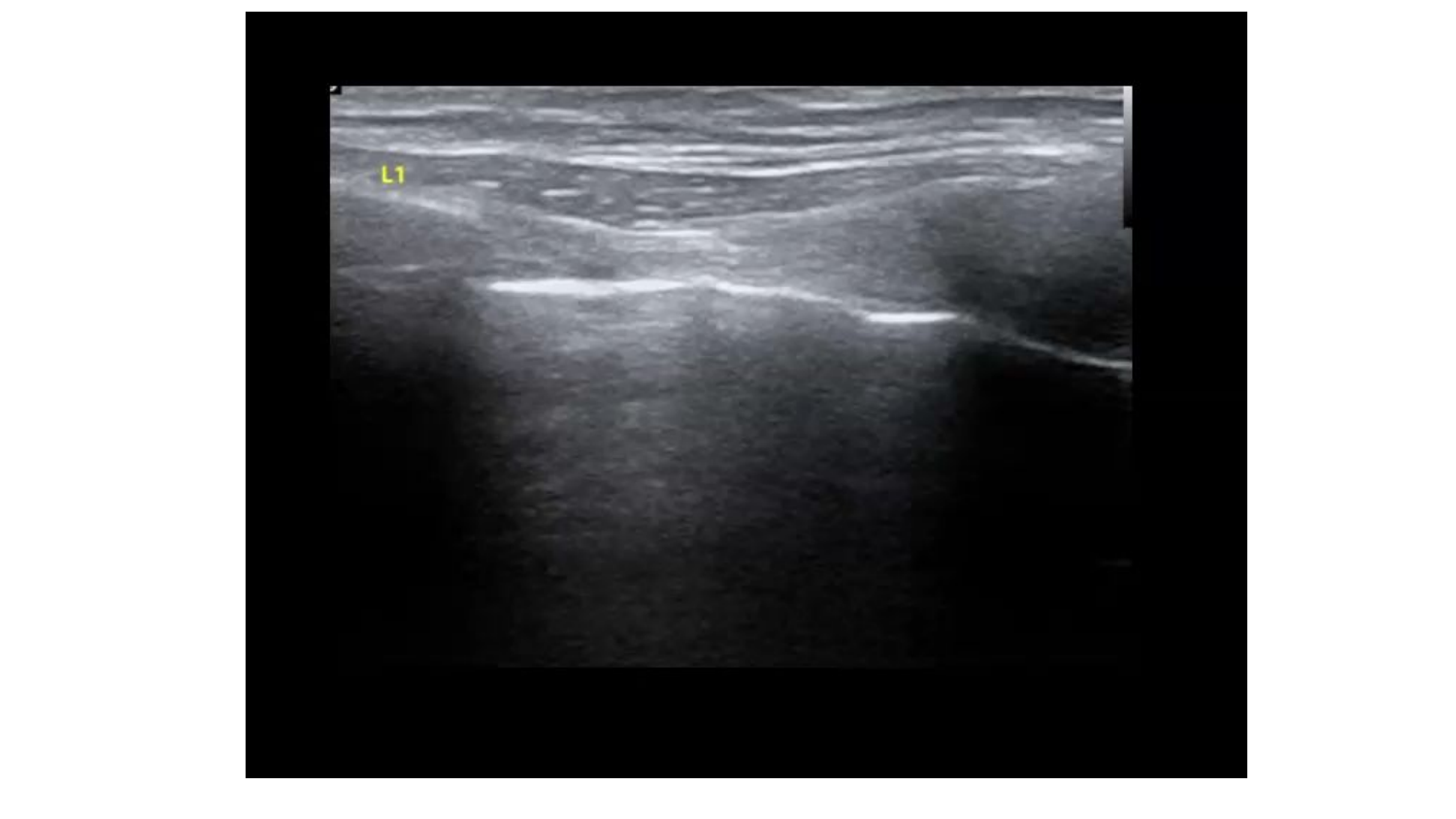

## Slide 9
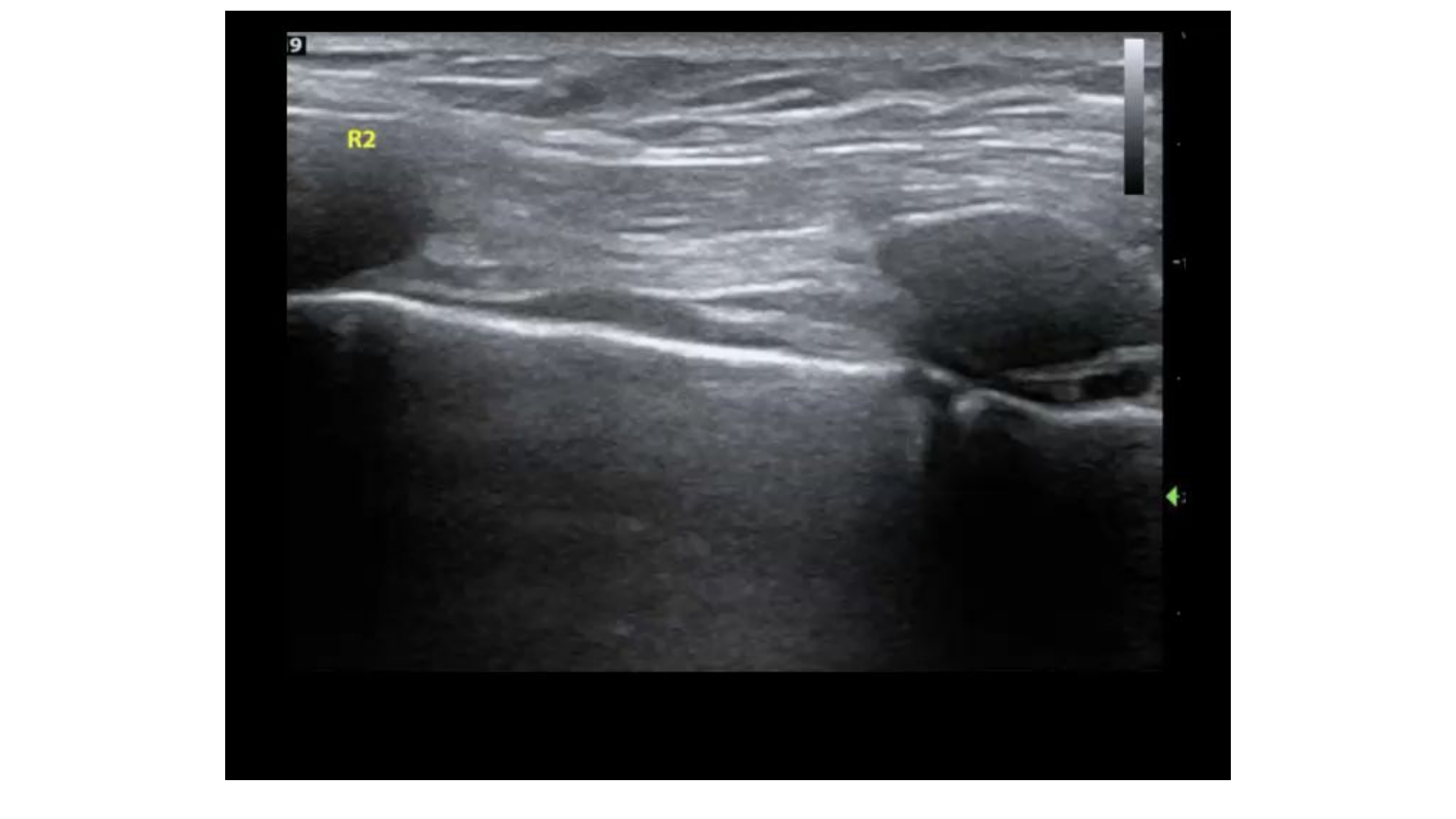

Supplement: Supplementary file 1 — Scenario 1.docxScenario 1 Setup and Prompts.docxScenario 1 Stimuli.pptxScenario 1 Skills Checklist.docxScenario 2.docxScenario 2 Setup and Prompts.docxScenario 2 Adult Stimuli.pptxScenario 2 Peds Stimuli.pptxScenario 2 Skills Checklist.docxScenario 3.docxScenario 3 Setup and Prompts.docxScenario 3 Skills Checklist.docxExample Schedule.xlsxDebriefing Material.docxPostsession Evaluation.docx [file mep_2374-8265.11361-s001.zip › G. Scenario 2 Adult Stimuli.pptx]

## Slide 1
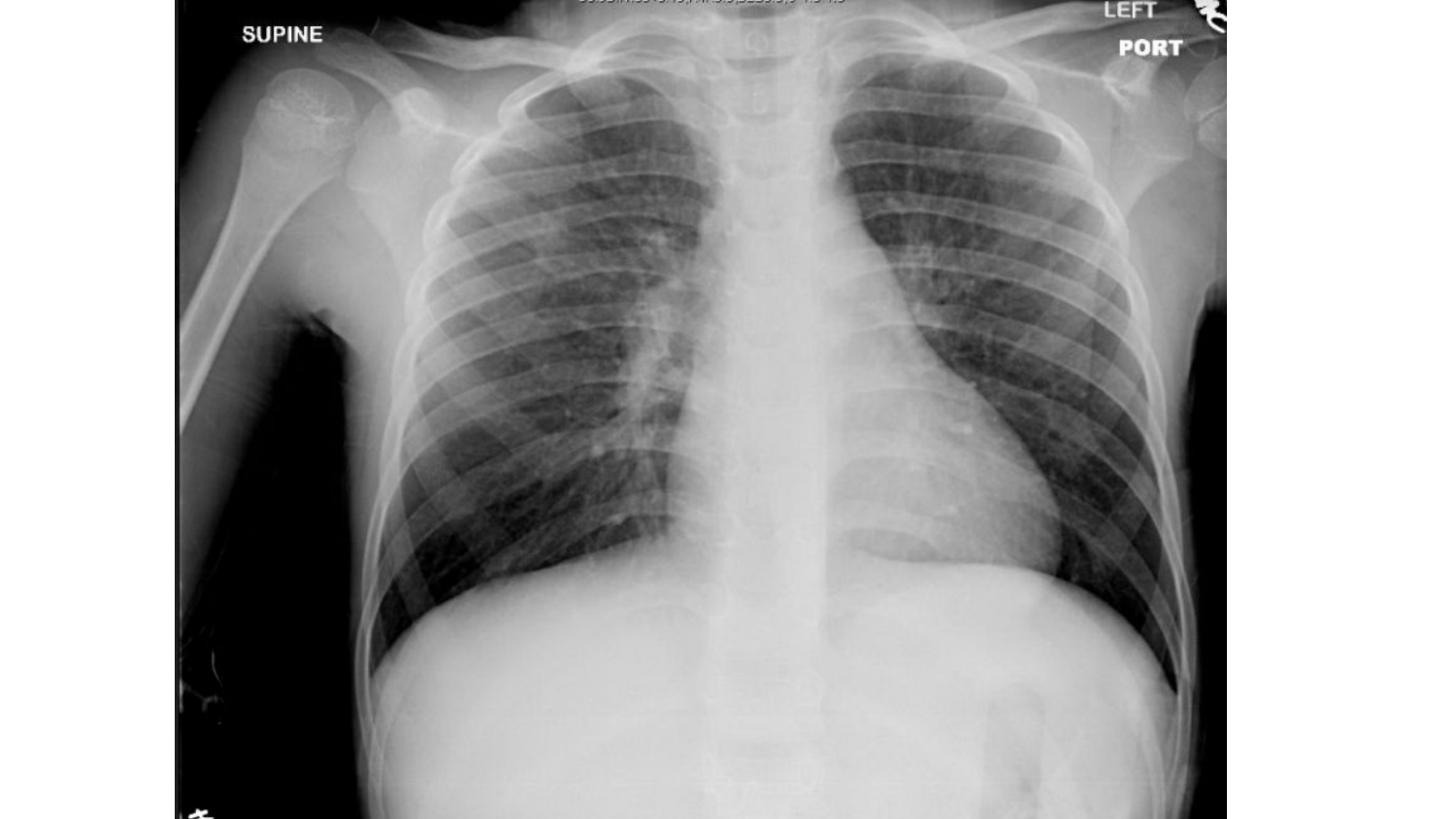

## Slide 2
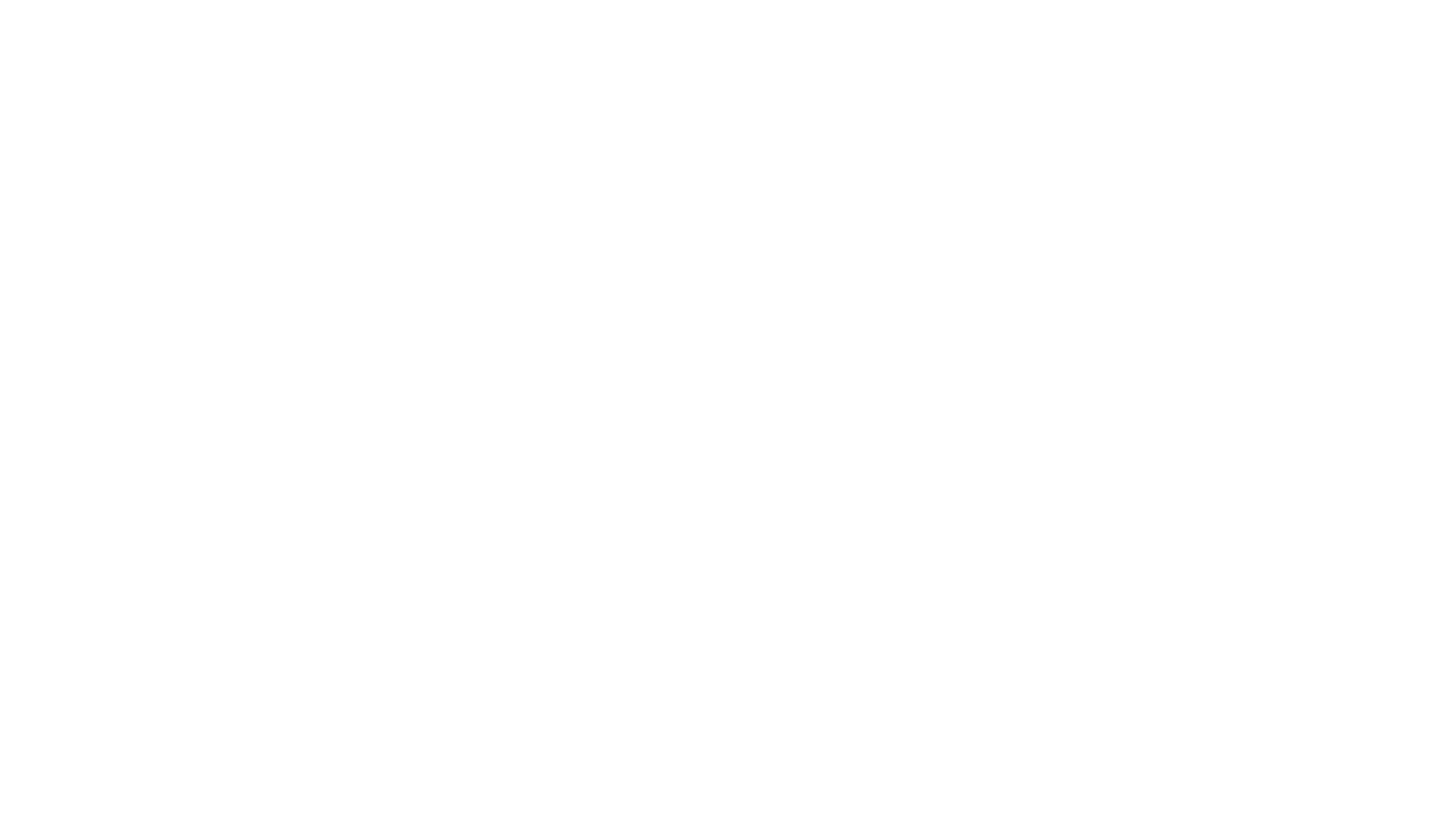

#

## Slide 3
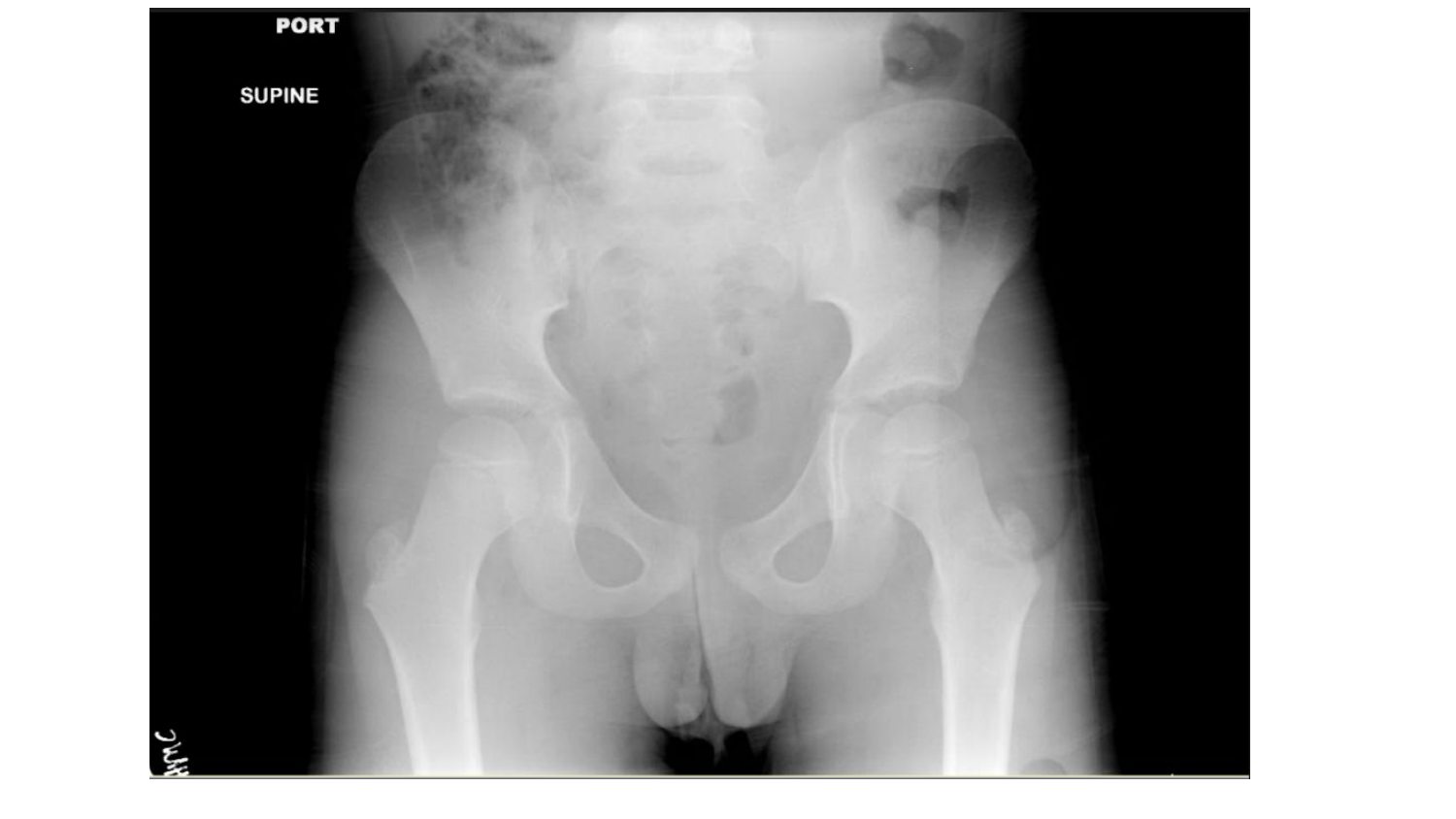

## Slide 4
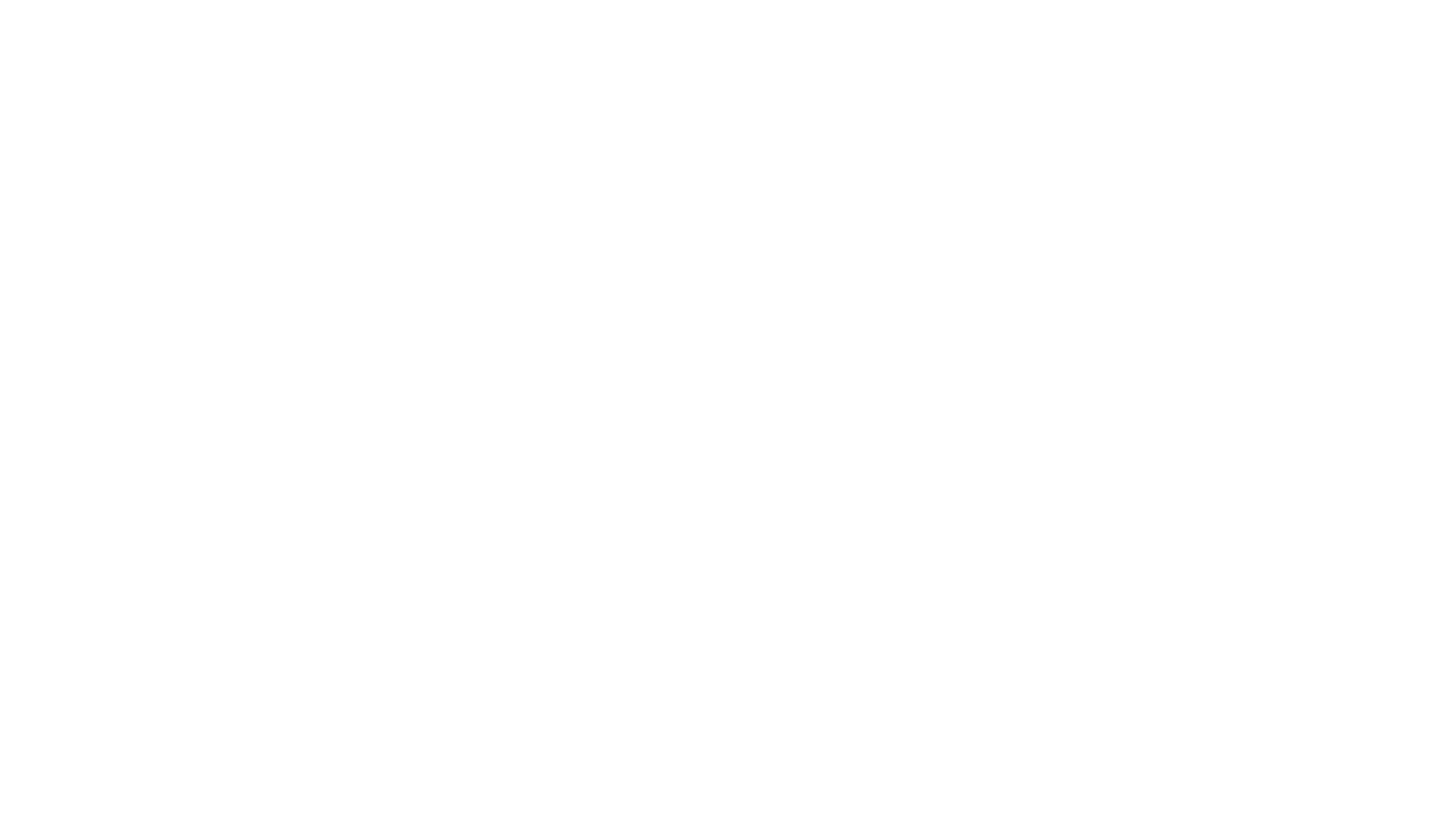

## Slide 5
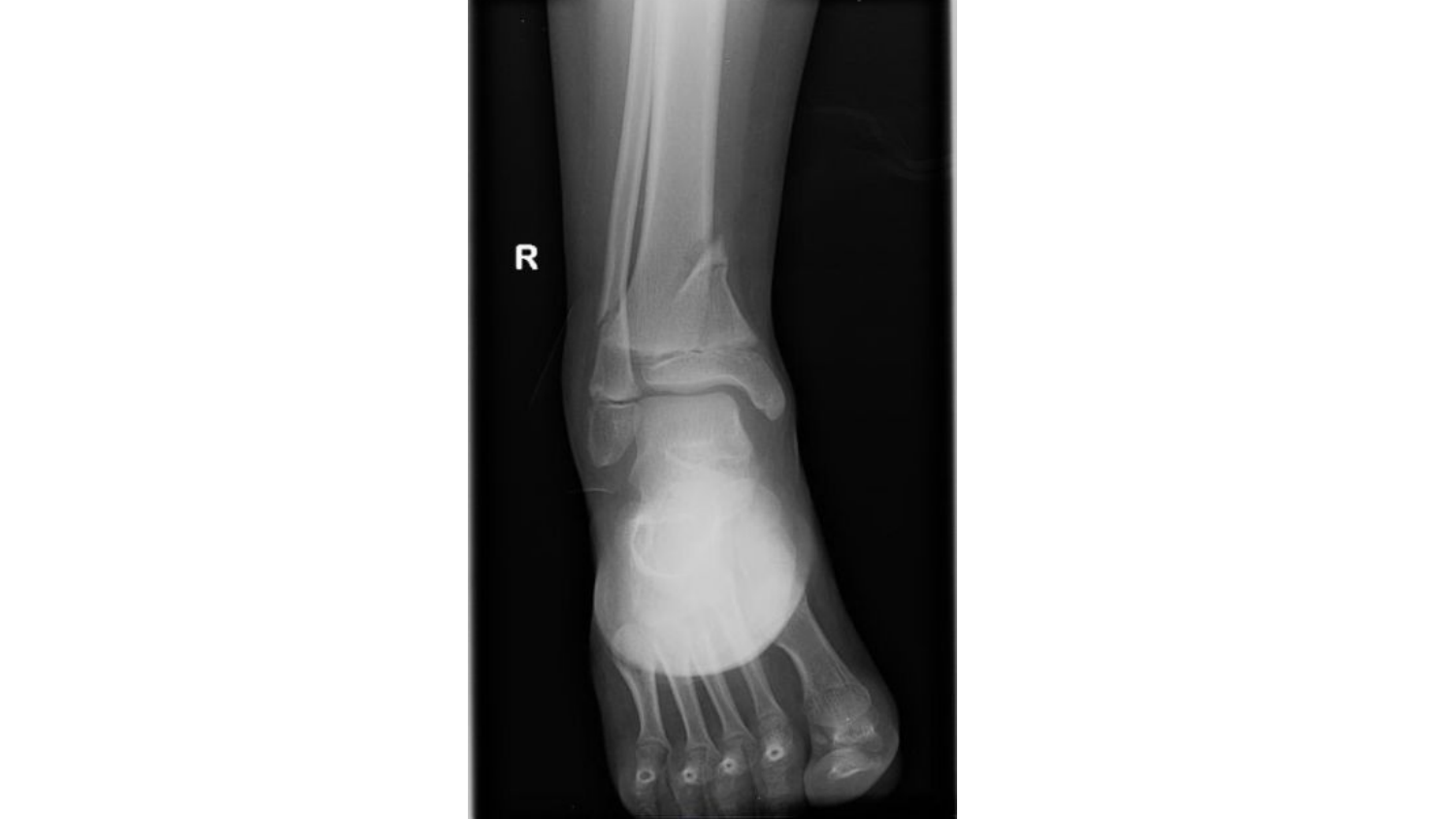

Supplement: Supplementary file 1 — Scenario 1.docxScenario 1 Setup and Prompts.docxScenario 1 Stimuli.pptxScenario 1 Skills Checklist.docxScenario 2.docxScenario 2 Setup and Prompts.docxScenario 2 Adult Stimuli.pptxScenario 2 Peds Stimuli.pptxScenario 2 Skills Checklist.docxScenario 3.docxScenario 3 Setup and Prompts.docxScenario 3 Skills Checklist.docxExample Schedule.xlsxDebriefing Material.docxPostsession Evaluation.docx [file mep_2374-8265.11361-s001.zip › H. Scenario 2 Peds Stimuli.pptx]
